# Comprehensive metabolomics analysis of prostate cancer tissue in relation to tumor aggressiveness and *TMPRSS2-ERG* fusion status

**DOI:** 10.1186/s12885-020-06908-z

**Published:** 2020-05-18

**Authors:** Ilona Dudka, Elin Thysell, Kristina Lundquist, Henrik Antti, Diego Iglesias-Gato, Amilcar Flores-Morales, Anders Bergh, Pernilla Wikström, Gerhard Gröbner

**Affiliations:** 1 Department of Chemistry, Umeå University, Linnaeus väg 6, 901 87 Umeå, Sweden; 2 Department of Medical Biosciences, Pathology, Umeå University, Umeå, Sweden; 3 IVS, Faculty of Health Sciences, University of Copenhagen, Copenhagen, Denmark; 4 Novo Nordisk Foundation Centre for Protein Research, Faculty of Health Sciences, University of Copenhagen, Copenhagen, Denmark; 5 Danish Cancer Society, Copenhagen, Denmark

**Keywords:** Metabolomics, Prostate cancer, *TMPRSS2-ERG*, ^1^H HRMAS NMR, Gleason score

## Abstract

**Background:**

Prostate cancer (PC) can display very heterogeneous phenotypes ranging from indolent asymptomatic to aggressive lethal forms. Understanding how these PC subtypes vary in their striving for energy and anabolic molecules is of fundamental importance for developing more effective therapies and diagnostics. Here, we carried out an extensive analysis of prostate tissue samples to reveal metabolic alterations during PC development and disease progression and furthermore between *TMPRSS2-ERG* rearrangement-positive and -negative PC subclasses.

**Methods:**

Comprehensive metabolomics analysis of prostate tissue samples was performed by non-destructive high-resolution magic angle spinning nuclear magnetic resonance (^1^H HR MAS NMR). Subsequently, samples underwent moderate extraction, leaving tissue morphology intact for histopathological characterization. Metabolites in tissue extracts were identified by ^1^H/^31^P NMR and liquid chromatography-mass spectrometry (LC-MS). These metabolomics profiles were analyzed by chemometric tools and the outcome was further validated using proteomic data from a separate sample cohort.

**Results:**

The obtained metabolite patterns significantly differed between PC and benign tissue and between samples with high and low Gleason score (GS). Five key metabolites (phosphocholine, glutamate, hypoxanthine, arginine and α-glucose) were identified, who were sufficient to differentiate between cancer and benign tissue and between high to low GS. In ERG-positive PC, the analysis revealed several acylcarnitines among the increased metabolites together with decreased levels of proteins involved in β-oxidation; indicating decreased acyl-CoAs oxidation in ERG-positive tumors. The ERG-positive group also showed increased levels of metabolites and proteins involved in purine catabolism; a potential sign of increased DNA damage and oxidative stress.

**Conclusions:**

Our comprehensive metabolomic analysis strongly indicates that ERG-positive PC and ERG-negative PC should be considered as different subtypes of PC; a fact requiring different, sub-type specific treatment strategies for affected patients.

## Background

Prostate cancer (PC) is one of the most prevalent cancers and a significant cause of morbidity and mortality in men [1]. This cancer comes in many flavors, since it is very heterogeneous in terms of grade, genetics, ploidy, and oncogene/tumor suppressor gene expression, and it displays complex biological, hormonal, and molecular features [2]*.* Moreover, this disease has diverse phenotypes ranging from indolent asymptomatic to very aggressive lethal forms [3]. Current diagnostic strategies are based on serum PSA levels and prostate biopsy histology, and have a very limited accuracy in predicting the clinical behavior of individual tumors, especially the ones prone to become aggressive at later stages. Therefore, precise risk classification is a central challenge in clinical PC research, and there is an urgent need for specific diagnostic tools to distinguish patients in terms of aggressiveness and choice of therapy; tools which would save the majority of PC patients unnecessary treatment with often severe side-effect [4].

Ever since the discovery of the genetic fusion between the erythroblast transformation-specific (*ETS*) transcriptional factor ETS-related gene (ERG) and the androgen-responsive promotor transmembrane protease, serine 2 (*TMPRSS2*) by Tomlins et al. [5], there has been an intense debate about its usefulness as biomarker for the detection and the stratification of PC [6]. The gene fusion *TMPRSS2-ERG* is the major genomic alteration found in about half of all PCs, and it leads to aberrant androgen dependent ERG expression [7]. *TMPRSS2-ERG* can already be found in low-score PC, and persists even in metastatic and castration-resistant types [8]. However, the debate is still ongoing if this molecular subtype displays distinct clinical and biological tumor characteristics. A majority of studies evaluating the potential of *TMPRSS2–ERG* in predicting PC aggressiveness, suggested that *TMPRSS2–ERG* is associated with aggressive or fatal PC, a shortened disease free survival period and an increase in PC specific death [9–11]. However, other studies failed to see any association between *TMPRSS2-ERG* and patient outcome [12, 13]. Nevertheless, some recent studies suggested metabolic alterations in *TMPRSS2-ERG*-positive PC [14, 15].

To differentiate different types of PC explicitly with respect to tumor grade and *TMPRSS2-ERG* status, we carried out a comprehensive metabolomics analysis on intact prostate tissue specimens to identify suitable metabolic markers. Metabolomics represents a powerful platform for extracting valuable information from sets of low-molecular weight metabolites, to provide a global understanding of pathophysiological alterations occurring during cancer progression [16]. In this study, we applied complementary analytical techniques; ^1^H HR MAS NMR on intact PC tissues, followed by liquid ^1^H NMR, ^31^P NMR spectroscopy and LC-MS on tissue extracts to explore metabolic alterations during PC development and disease progression from lower to higher GS and between *TMPRSS2-ERG*-positive and -negative PC. Analysis of the metabolomics data by advanced chemometrics based bioinformatics enabled us to identify biomarkers of potential high diagnostic value; and these markers provided a better molecular understanding of PC biology in relation to tumor de-differentiation as well as *TMPRSS2-ERG* fusion gene expression. The novel molecular knowledge obtained here will be highly valuable for developing specific PC diagnostics and subtype-specific therapies.

## Materials

### Patients and tissue samples specimens

Fresh-frozen prostate tissues were selected from a series of samples collected from patients who underwent radical prostatectomy at Urology Clinic at Umeå University Hospital between 2009 and 2012. The patients gave written informed consent and the ethical committee for Umeå University approved the use of these samples for research. Immediately after surgical removal the prostates had been brought to the Pathology Department and cut in 0.5 cm thick slices. From these slices 20 samples were punched using a 0.5 cm steel cylinder and frozen in − 70 °C within 30 min after surgery. The prostate slices were then fixed in 4% formaldehyde for 24 h, dehydrated, embedded in paraffin (FFPE), cut in 5 μm thick sections and stained with hematoxylin-eosin (H&E). Frozen samples from 16 patients were carefully selected based on the histopathology of the FFPE sections [17] to include non-malignant and malignant tissues, and at the end those were successfully isolated from 13 and 14 cases, respectively. Each frozen biopsy was cut into 2 to 6 replicates, resulting in altogether 129 samples that were stored in − 80 °C. After ^1^H HR MAS NMR spectroscopy and metabolite extraction, samples were transferred to Molecular Fixative (UMFix, Sakura, Torrance, CA, USA) and further processed for histology examination. The tissue samples were cut in 5 μm thick sections using a cryostat. Detailed histopathological assessment was carried out to determine the relative fraction (percentage) of epithelial and stromal tissue, the fraction of malignant cells, and the tumor differentiation according to the Gleason grade scale using cryostat sections immunostained for high molecular weight cytokeratin (HMW-CK, Dako, Stockholm, Sweden) and PAN-CK (AE1/AE3, Dako), as previously described [17]. Briefly, the percentage of tumor tissue (glands lacking HMW-CK positive basal epithelial cells) and non-malignant tissue (glands with an intact basal epithelial cell layer) and the tumor Gleason score were determined for all sections as follows. The fraction of malignant vs. non-malignant tissue in each sample was determined by using a light microscope with a square-lattice mounted in the eye-piece to count the number of grid-intersections falling on each tissue compartment. The Gleason score (GS) was determined by one pathologist (A.B.) and expressed as the primary plus secondary Gleason grades.

The *TMPRSS2-ERG* status was accessed by immunohistochemical ERG-staining [11]. Ten tissue samples were embedded in Optimal Cutting Temperature (OCT) solution before cryo-sectioning and therefore not used for ^1^H HR MAS NMR analysis. The clinical sample characteristics are summarized in Table 1. Because of observed heterogeneity, each replicate was treated as an individual sample in the metabolic analysis.

**Table 1:** Patient and sample characteristics

	Total number	Total number in ERG-negative	Total number in ERG-positive
**Patients**	16	7	10
**Benign samples**	59		
**Malignant samples**			
all	70	24	46
OCT embedded	10	6	4
not-OCT embedded	60	18	42
**Percentage of epithelium**			
1–25%	24	6	18
26–50%	39	15	24
51–75%	6	3	3
> 75%	1	0	1
**Percentage of malignancy**			
1–10%	21	3	18
11–20%	24	7	17
21–30%	10	7	3
31–40%	8	4	4
41–50%	3	3	0
51–60%	0	0	0
61–70%	4	0	4
**Percentage of malignancy to total epithelium area**			
1–25%	9	1	8
26–50%	18	5	13
51–75%	16	7	9
> 75%	27	11	16
**Gleason score**			
3 + 3	43	7	36
3 + 4	16	10	6
4 + 3	10	6	4
4 + 4	1	1	0

### ^1^H HR MAS NMR analysis of intact tissues

Tissue samples were thawed at room temperature and kept on ice at all times during the preparation process to minimize metabolite degradation. Each tissue sample (30–50 mg wet weight) was inserted into disposable 30-μL teflon NMR inserts followed by the addition of ∼10 μ D_2_O. Inserts were transfered into 4 mm zirconia MAS rotors and NMR spectra were obtained at 283 K on 500 MHz NMR spectrometer (Bruker Biospin, Karlsruhe, Germany). ^1^H HR MAS NMR spectra were acquired and processes as described previously [17, 18] using a 1D Carr-Purcell- Meiboom-Gill (CPMG) spin-echo pulse sequence and a sample spinning rate of 5 kHz. The proton chemical shifts were referenced to CH_3_ signal of lactate at 1.33 ppm. Phased and baseline corrected CPMG spectra were converted into statistical matrices using Chenomix v.7.72 (Chenomx Inc., Edmonton AB, Canada). Spectra were divided and signal integrals were computed in δ0.04 intervals. Each integrated NMR spectral region was normalized to total intensity. Metabolite identification and chemical assignment were performed on the basis of the literature and with application of Chenomix.

### Metabolite extraction from intact tissues

A sample extraction protocol was used as described by Brown et al. [19] with small modifications. Briefly, after ^1^H HR MAS NMR experiments the tissue sample was immediately removed from the NMR rotor and immediately placed in cryo-vials containing 5 ml of solvent (80% methanol, 20% ultra-pure water). Samples were incubated for 24 h at room temperature. Thereafter, the intact tissue sample was separated from the solvent extract and processed for histological investigations as described in detail below. The solvent extract was spun for 5 min at 2000 rpm, and the supernatant was evaporated to dryness under a stream of nitrogen gas. The dried extracts were reconstituted in 600 μl of deuterated methanol: deuterated water (80:20 vol/vol) containing LC standards: Caffeine (trimethyl-^13^C_3_), Cholic Acid (2,2,4,4-D_4_), Arachidonic Acid-D_8_, Caffeic Acid-^13^C_9_. Following metabolite extraction, samples were stored at − 80 °C until further analysis.

### ^31^P NMR analysis of tissue extracts

Measurements were performed at 298 K on a 31P direct observe 5 mm BBO cryoprobe on a 600 NMR spectrometer (Bruker, Fällanden, Switzerland). Spectra were recorded using 1400 scans and the spectral width of 15,000 Hz. Spectra were processed using TopSpin software v.3.2 and 1.0 Hz line broadening was applied. Phosphatidylcholine, the most common and highest concentrated phospholipids, was used for calibration (− 0.84 ppm). All peaks in the NMR spectra were integrated by in-house Matlab script (R2015a) and normalized to total intensity. The assignment of resonances was performed with the aid of chemical shift values reported in the literature [20, 21]. Sixteen phospholipids were detected according to their specific chemical shift values.

### ^1^H NMR analysis of tissue extracts

NMR spectra were recorded on a Bruker 600 NMR spectrometer (Bruker, Fällanden, Switzerland) at 298 K. To acquire ^1^H NMR spectra of tissue extracts a standard CPMG pulse sequence was used to suppress broad signals arising from macromolecules. The 90^o^ pulse was set to 10 μs, and 128 scans were acquired into 64 k data points using a spectral width of 7200 Hz (12 ppm). The obtained FID was processed as described above and chemical shifts referenced to CH_3_ signal of lactate at 1.33 ppm. Spectra were imported into MATLAB (R2015a), integrated using in-house developed scripts and normalized by the sum of all intensities. Peak assignments were carried out as described for ^1^H HR MAS NMR.

### LC-MS analysis of tissue extracts

Untargeted metabolite profiling was carried using UHPLC-QTOFMSMS (Agilent 6540) equipped with a Kinetics 2.1 × 100 1.7u C18 column in positive and negative mode. The injection volume was 1 μL and column oven temperature was set to 40 °C. Samples were analyzed by a 11 min revered-phase chromatography with gradient elution at 0.5 min/min flow rate from 99% mobile phase H_2_O (0.1% formic acid) to 99% mobile acetonitrile (0.1% formic acid). The order of injection of samples was randomized. QC samples were used to monitor the performance of UPLC-MS system, and were run at the beginning of the run (to condition the chromatographic column) and periodically after every 10 samples. Analyses were conducted separately for positive and negative modes. Two solvent blanks were injected at the end of each run to identify any features introduced from the extraction process and solvent systems.

Data processing was done in Profinder v. B.06.00 (Agilent Technologies Inc., Santa Clara, CA, USA). Targeted feature extraction (TFE) was applied and as an input formula source an in-house reference library (Swedish Metabolomics Centre) [22], composed of 713 authentic chemical standard entries that included retention time, molecular weight (m/z), preferred adducts, and in-source fragments as well as their associated MS/MS2 spectra. Only peaks found in all subjects and identified were used in the analysis. The processed data set thus consisted of 70 samples characterized by 66 variables (identified metabolite peaks) in positive mode and 81 variables (identified metabolite peaks) in negative mode. For selected metabolite biomarkers structural assignments were also carried out by matching MS/MS spectra, to tandem MS experiments from on-line databases and in-house databases (Swedish Metabolomics Centre) [22]. Since it was not the scope of this work to fully identify all individual metabolites the acquired tentative not significant identities were not further analyzed or confirmed. LC/MS data was normalized total ion counts which relate ion counts under a given peak to total ion counts.

### Univariate and multivariate analysis

Processed ^1^H HR MAS NMR, ^1^H NMR, ^31^P NMR and LC-MS data were subjected to both univariate and multivariate analyses. The processed data sets were UV-scaled prior to multivariate analysis in SIMCA-P+ (version 13.03, Umetrics, Umeå, Sweden). Principal component analysis was used for unsupervised variation analysis to detect groups and trends in the data and orthogonal partial least squares discriminant analysis (OPLS-DA) was applied as a supervised means to identify the discriminating metabolites between selected sample groups. Analysis of variance of cross-validated predictive residuals (CV-ANOVA) was used to assess the significance of the OPLS-DA models. The *p*-value obtained from this analysis indicates the probability level that a given model has been built by chance, and a *p*-value lower than 0.05 is associated with a significant model. Using a combination of loadings following OPLS-DA, the most perturbed metabolites between selected groups were determined. The differential metabolites were additionally validated by nonparametric *t*-test with Benjamini–Hochberg multiple testing correction performed using in-house software written and compiled in MATLAB (Mathworks). GraphPad Prism 6 (San Diego, CA, U.S.A.) was used to calculate the average of metabolite levels, which were expressed as mean ± SEM.

### Mixed models

The processed data were further evaluated using linear mixed models in order to account for repeated measures. In brief, linear mixed models contain additional random effect terms (in this case the individuals having repeated samples) compared to standard linear models. Furthermore, adjusted linear mixed models were constructed; adjusting for epithelium, malignancy, ratio of malignancy to total epithelium and GS. In the adjusted models the adjusting factors, eg. Gleason score, enters the model as a covariate in a similar fashion as for normal linear regression. In order to correct for multiple testing Benjamini-Hochberg corrected *p*-values (ie. q-values) were calculated. Prior to modelling the ^1^H NMR and LC-MS data was subjected to different transformation methods, eg. log2, square root or the inverse of square root, according to histograms, qq-plots and the Shapiro-Wilk test of normality for the residuals. The transformation, modelling and multiple testing correction was performed in the free software environment R version 3.3.2 (https://www.R-project.org/) and the R-package nlme (http://CRAN.R-project.org/package=nlme).

### Analysis of proteomic data

The proteomic analysis with LC-MS/MS on a Q-Exactive mass spectrometer had been previously performed in a separate patient cohort including prostatectomy samples [23]. The cohort was annotated according to ERG rearrangement (ERG-immunostaining) and contain 12 ERG-positive and 16 ERG-negative PC cases.

## Results

**Fig. 1:**
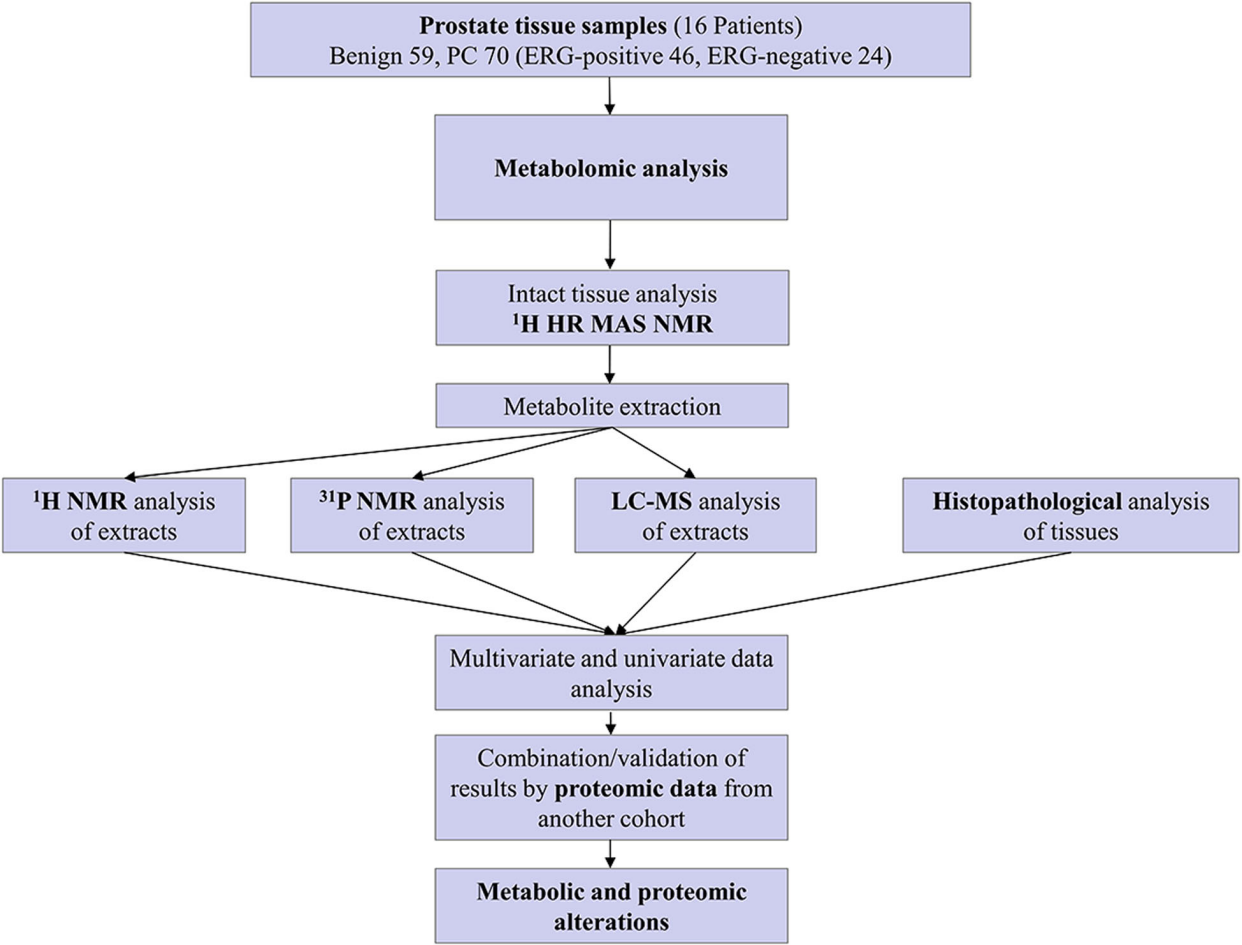
A flowchart depicting the outline of the study. Workflow and steps evolved for the metabolomic study conducted on tissue samples using ^1^H HR MAS NMR, ^1^H NMR, ^31^P NMR data and LC-MS (+/−) approaches are shown

This study included in total 129 prostate tissue samples, obtained as replicates from radical prostatectomy specimens from 16 PC patients (Table 1). To increase the information content from each sample, a workflow scheme (Fig. 1) was developed for enabling complementary metabolomics analysis and histological evaluation of the same tissue sample. Metabolomic profiles of intact tissue specimens were acquired by ^1^H HR MAS NMR, followed by subsequent analysis of tissue extracts originating from the original specimens by ^1^H NMR, ^31^P NMR spectroscopy and LC-MS without any need for exchange of solvents due to the use of deuterated solvents. This mild extraction approach allowed subsequent histological evaluation since the tissue morphology remained intact (Fig. 1). Clinical and histological characteristics of the patients and their tissue samples are summarized in Table 1. Altogether, 136 metabolites including different lipid species were identified (summary see Additional file 1: Table S1). We observed, as others [24], variation in the total amount of metabolites in the extracted tissues most likely related to sample size and composition of tissues. Therefore, we used relative intensities of metabolites in all data sets. For LC/MS data these relative intensities reference to the peak height of the individual metabolites in relation to total ion counts in the sample; and for all NMR-data sets the relative intensities relate to the integral of the individual metabolites in relation the sum of all integrals for each spectrum. Our NMR-based profiles were used as control for sample variability by comparing common metabolites in NMR and LC/MS data sets. Furthermore, we applied normalization methods, for LC/MS data – normalization to total ion counts and for all NMR-data sets normalization by the sum of all intensities, and strict selection of biomarkers to overcome this problem.

### Analysis of PC and adjacent benign prostate

**Fig. 2:**
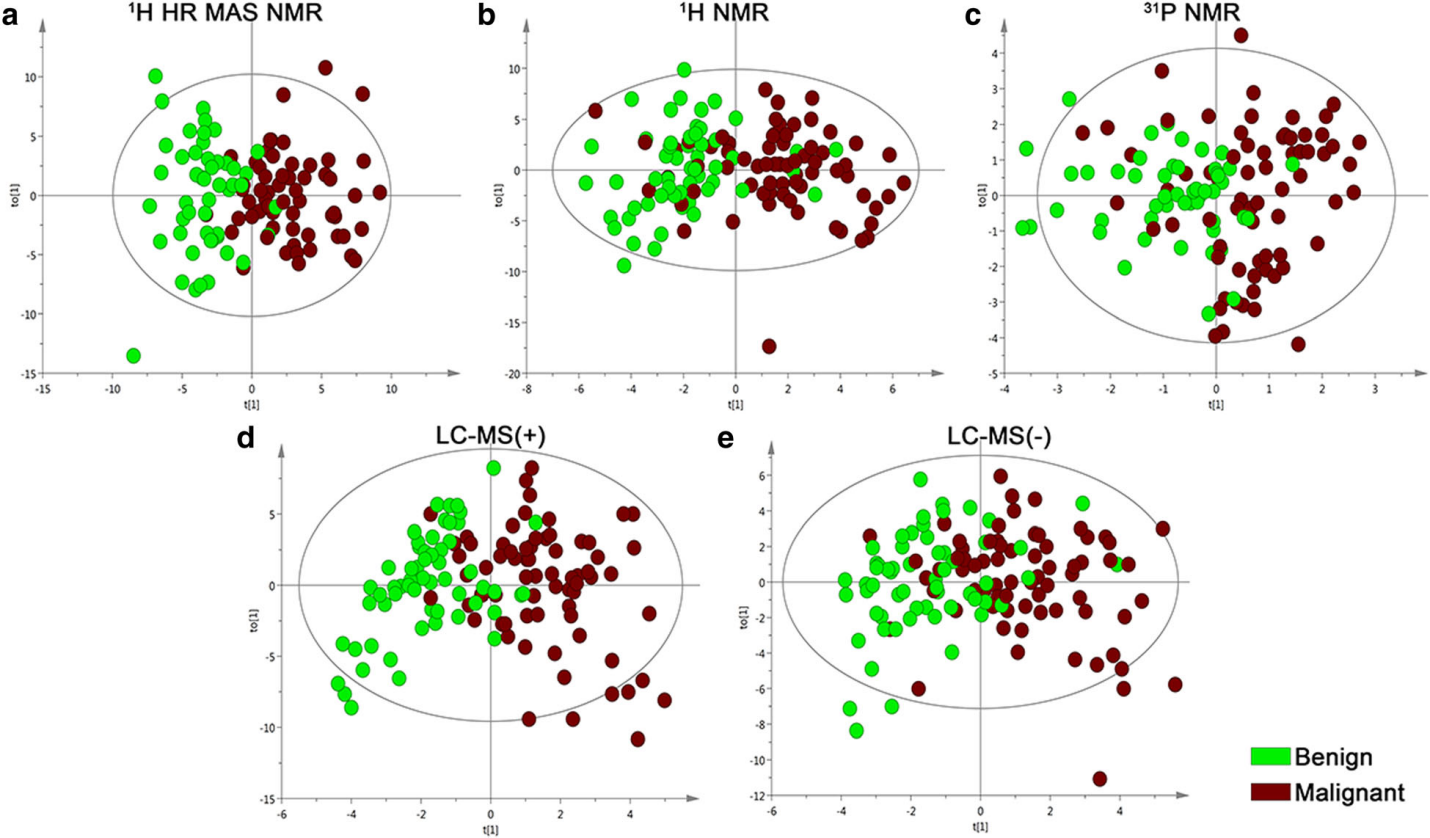
Tissue metabolomics multivariate analysis of prostate cancer. OPLS-DA score plots of benign samples (green dots) and malignant samples (brown dots) **a**. ^1^H HR MAS NMR data, **b**. ^1^H NMR data, **c**. ^31^P NMR data, **d**. LC-MS (+) data, **e**. LC-MS (−) data

Initially, principal component analysis was applied in an unsupervised variation analysis of the data originating from ^1^H HR MAS NMR, ^1^H NMR ^31^P NMR and LC-MS (positive and negative mode). The corresponding principal component analysis score plots (Additional file 2: Figure S1A-E) display a clear trend of clustering of the malignant and normal samples, respectively. To maximize the sample group separation and identify discriminating metabolites, supervised discrimination models were established based on orthogonal partial least squares discriminant analysis (OPLS-DA), and a clear class discrimination was obtained for each of the data sets (Fig. 2a-e). Goodness of fit values and predictive ability values (R2X, R2Y, Q2) were obtained (Additional file 3: Table S2), indicating that all models possessed a reasonable fit and predictive power. A CV-ANOVA test showed highly significant variation related to the separation of groups (Additional file 3: Table S2). Validation plots confirmed the robustness of the OPLS-DA models (Additional file 4: Figure S2A-E). Table 2 shows the identity of the features in the OPLS-DA models that significantly discriminated between PC and adjacent benign prostate tissues.

**Table 2:** Metabolic alterations in prostate cancer tissues compared to benign prostate tissues

Metabolite	Change in PC	Technique	BH *p*-value^a^	BH *p*-value for mixed model	BH *p*-value for mixed model adjusted for percentage of epithelium	BH *p*-value for mixed model adjusted for percentage of malignancy	BH *p*-value for mixed model adjusted for percentage of epithelium and malignancy
Phosphocholine	↑	^1^H HR MAS NMR	1.08 × 10^− 07^	2.53 × 10^− 10^	2.81 × 10^− 08^	1.22 × 10^− 03^	1.24 × 10^− 04^
Glutamate	↑	^1^H HR MAS NMR	7.17 × 10^− 07^	2.26 × 10^− 08^	6.41 × 10^− 07^	5.91 × 10^− 03^	3.87 × 10^− 04^
Citrate	↓	^1^H HR MAS NMR	6.66 × 10^− 06^	6.48 × 10^− 08^	1.97 × 10^− 07^	1.44 × 10^−02^	2.07 × 10^− 02^
Hypoxanthine	↑	^1^H HR MAS NMR	4.68 × 10^−08^	1.58 × 10^−08^	1.11 × 10^−05^	5.91 × 10^− 03^	7.73 × 10^− 05^
Polyamines	↓	^1^H HR MAS NMR	9.61 × 10^− 05^	6.16 × 10^− 08^	1.42 × 10^−07^	3.06 × 10^− 03^	1.22 × 10^− 02^
Inosine	↑	^1^H HR MAS NMR	1.35 × 10^− 05^	1.07 × 10^−04^	5.06 × 10^− 03^	7.25 × 10^− 02^	1.02 × 10^− 02^
α-Glucose	↓	^1^H NMR	8.89 × 10^− 04^	1.58 × 10^− 08^	2.09 × 10^− 05^	1.75 × 10^− 02^	1.24 × 10^− 04^
Nicotinamide adenine dinucleotide (NAD^+^)	↓	^1^H NMR	3.17 × 10^− 03^	3.81 × 10^− 07^	7.77 × 10^−06^	3.06 × 10^− 03^	1.87 × 10^− 04^
Arginine	↑	^1^H NMR	8.89 × 10^− 04^	2.98 × 10^− 06^	5.46 × 10^− 02^	3.77 × 10^− 01^	1.41 × 10^− 04^
Succinate/Malate	↑	^1^H NMR	2.40 × 10^− 02^	6.80 × 10^− 03^	6.67 × 10^− 01^	4.38 × 10^− 01^	1.80 × 10^− 03^
Lysophosphatidylcholine	↓	^31^P NMR	1.44 × 10^− 04^	1.87 × 10^− 02^	3.16 × 10^− 01^	6.53 × 10^− 01^	4.31 × 10^− 02^
Phosphatidylethanolamine	↑	^31^P NMR	5.50 × 10^− 03^	4.82 × 10^− 04^	1.49 × 10^− 03^	1.05 × 10^− 02^	5.87 × 10^− 03^
Sphingomyelin	↓	^31^P NMR	1.01 × 10^− 03^	4.22 × 10^− 05^	2.12 × 10^− 04^	8.34 × 10^− 03^	3.20 × 10^− 03^
Uracil	↑	LC-MS (+)	9.69 × 10^− 05^	2.98 × 10^− 06^	3.59 × 10^− 05^	1.71 × 10^− 02^	1.42 × 10^− 03^
Docosapentaenoic acid (22:5)	↑	LC-MS (−)	5.01 × 10^− 05^	3.81 × 10^− 07^	3.38 × 10^− 04^	1.44 × 10^− 02^	7.73 × 10^− 05^
Oleic acid (18:1)	↑	LC-MS (−)	9.32 × 10^− 05^	3.52 × 10^− 06^	1.09 × 10^− 03^	4.22 × 10^− 03^	9.63 × 10^− 05^
Linoleic acid (18:2)	↑	LC-MS (−)	2.29 × 10^− 04^	1.45 × 10^− 05^	3.14 × 10^− 03^	1.05 × 10^− 02^	1.53 × 10^− 04^
Docosahexaenoic acid (22:6)	↑	LC-MS (−)	1.25 × 10^− 03^	9.69 × 10^− 04^	7.92 × 10^− 02^	2.86 × 10^− 02^	7.66 × 10^− 04^
Maleic acid	↑	LC-MS (−)	9.32 × 10^− 05^	5.63 × 10^− 07^	2.96 × 10^− 05^	2.71 × 10^− 01^	6.19 × 10^− 02^
Malic acid (Fumarate)	↑	LC-MS (−)	2.92 × 10^− 03^	6.86 × 10^− 03^	3.20 × 10^− 02^	2.71 × 10^− 01^	3.90 × 10^− 02^

*BH* Benjamini–Hochberg multiple testing correction; ^a^ nonparametric *t*-test

### Analysis of high-score versus low-score PC

**Fig. 3:**
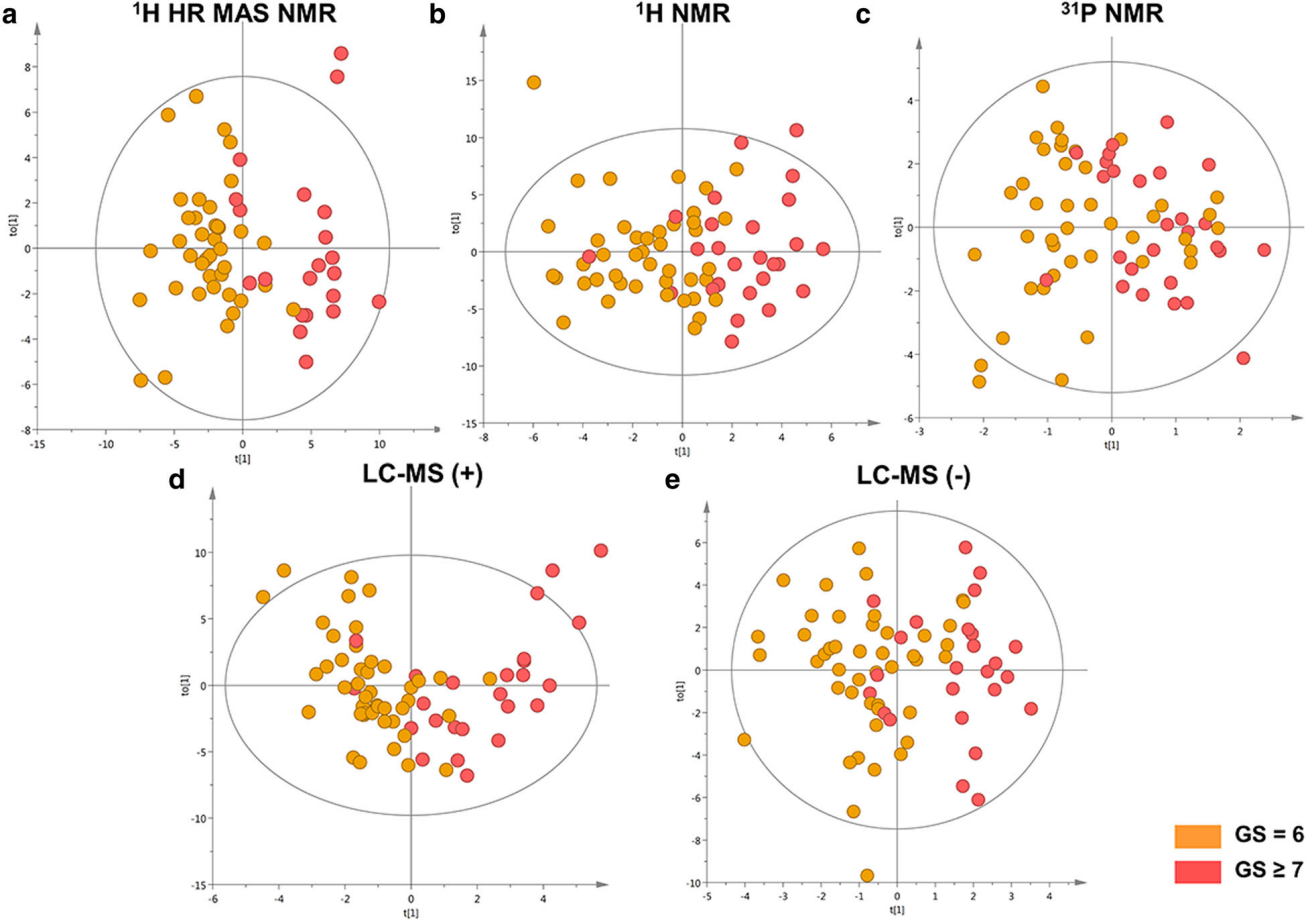
Tissue metabolomics multivariate analysis of Gleason scores. OPLS-DA score plots of Gleason score = 6 samples (orange dots) and Gleason score ≥ 7 samples (red dots) **a**. ^1^H HR MAS NMR data, **b**. ^1^H NMR data, **c**. ^31^P NMR data, **d**. LC-MS (+) data, **e**. LC-MS (−) data

In the multivariate models separating PC tissues from adjacent normal prostate tissues, we also observed patterns related to tumor differentiation, i.e. GS. Therefore OPLS-DA models were used to identify metabolites which differentiated between high-score PC, defined as GS 3 + 4, 4 + 3 or 4 + 4 (GS ≥ 7), and low-score PC, defined as GS 3 + 3 (GS = 6). A good separation of PC samples in relation to GS was obtained by ^1^H HR MAS NMR data on intact tissues, and ^1^H NMR/LC-MS (+) data on extracts (see Fig. 3a, b, d; Additional file 3: Table S2). However, based on the ^31^P NMR (Fig. 3c) and LC (−) (Fig. 3) data, samples were not significantly separated with respect to GS. Additionally, principal component analysis score plots are shown for each data set in Additional file 5: Figure S3A-E. Validation plots of the OPLS-DA models are presented in Additional file 6: Figure S4A-E. In Table 3 the metabolites that significantly differed between high and low GS samples based on univariate and multivariate analysis are shown.

**Table 3:** Metabolic alterations in high Gleason score (GS ≥ 7) to low Gleason score (GS = 6) prostate cancer

Metabolite	Change in GS ≥ 7	Technique	BH *p*-value^a^	BH *p*-value for Mixed Model	BH *p*-value for Mixed Model adjusted for percentage of epithelium	BH *p*-value for Mixed Model adjusted for percentage of malignancy	BH *p*-value for Mixed Model adjusted for percentage of epithelium and malignancy
Glycerophosphorylcholine	↑	^1^H HR MAS NMR	1.89 × 10^−04^	4.46 × 10^− 2^	2.94 × 10^− 1^	1.06 × 10^− 1^	2.07 × 10^− 2^
Phosphocholine	↑	^1^H HR MAS NMR	3.10 × 10^− 03^	1.91 × 10^− 2^	4.23 × 10^− 2^	8.04 × 10^− 2^	2.07 × 10^− 2^
Hypoxanthine	↑	^1^H HR MAS NMR	2.29 × 10^− 05^	3.51 × 10^− 4^	5.37 × 10^− 2^	9.01 × 10^− 2^	7.81 × 10^− 4^
Lysine	↑	^1^H HR MAS NMR	1.89 × 10^− 04^	3.04 × 10^− 4^	4.43 × 10^− 2^	6.54 × 10^− 2^	2.68 × 10^− 4^
Glutamate	↑	^1^H HR MAS NMR	2.95 × 10^− 05^	1.28 × 10^− 4^	2.30 × 10^− 2^	7.86 × 10^− 2^	3.33 × 10^− 4^
Threonine	↑	^1^H HR MAS NMR	5.95 × 10^− 03^	4.34 × 10^− 2^	4.17 × 10^− 1^	3.28 × 10^− 1^	2.07 × 10^− 2^
Tyrosine	↑	^1^H HR MAS NMR	9.99 × 10^− 03^	3.97 × 10^− 3^	4.71 × 10^− 2^	6.54 × 10^− 2^	1.72 × 10^− 3^
Valine	↑	^1^H HR MAS NMR	6.90 × 10^− 03^	3.64 × 10^− 3^	4.23 × 10^− 2^	6.54 × 10^− 2^	3.54 × 10^− 3^
Ascorbate	↑	^1^H HR MAS NMR	4.26 × 10^− 03^	1.78 × 10^− 2^	2.57 × 10^− 1^	3.44 × 10^− 1^	2.07 × 10^− 2^
Phenylalanine	↑	^1^H HR MAS NMR	4.34 × 10^− 02^	3.52 × 10^− 2^	5.37 × 10^− 2^	9.01 × 10^− 2^	2.07 × 10^− 2^
α-Glucose	↓	^1^H NMR	2.25 × 10^− 02^	2.02 × 10^− 8^	3.02 × 10^− 3^	1.42 × 10^− 1^	2.68 × 10^− 4^
Arginine	↑	^1^H NMR	2.44 × 10^− 03^	2.02 × 10^− 8^	5.37 × 10^− 2^	9.01 × 10^− 2^	6.36 × 10^− 7^
Lipid (*n*) CH_2_	↓	^1^H NMR	2.25 × 10^− 02^	4.34 × 10^− 2^	9.18 × 10^− 1^	6.14 × 10^− 1^	4.07 × 10^− 3^
2-Hydroxybutyrate	↑	^1^H NMR	2.44 × 10^− 03^	1.39 × 10^− 3^	5.76 × 10^− 1^	1.42 × 10^− 1^	1.56 × 10^− 4^
Sphingosine	↑	LC-MS (+)	5.62 × 10^− 03^	2.73 × 10^− 1^	6.49 × 10^− 1^	6.75 × 10^− 1^	3.06 × 10^− 1^
Hexanoylcarnitine	↑	LC-MS (+)	5.62 × 10^− 03^	2.10 × 10^− 1^	3.53 × 10^− 1^	3.28 × 10^− 1^	2.37 × 10^− 1^

*BH* Benjamini–Hochberg multiple testing correction; ^a^ nonparametric *t*-test

### Integration of metabolomic data related to PC and tumor differentiation

**Fig. 4:**
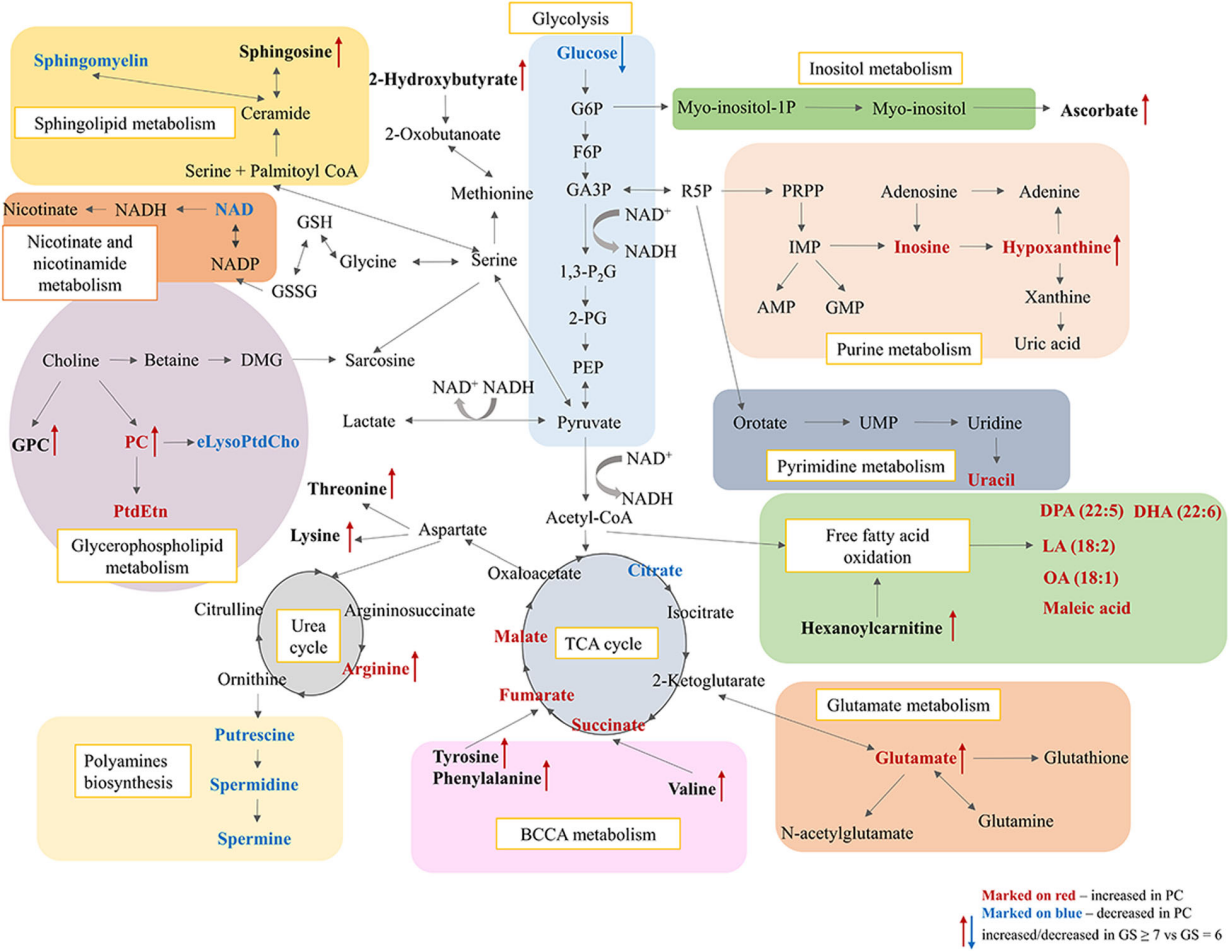
Metabolomics pathway network map of significantly altered metabolites in prostate cancer compared to benign prostate and additionally high Gleason score compared to low Gleason score. Metabolites significantly increased in PC are marked on red, significantly decreased in PC are marked on blue. Metabolites significantly increased and decreased in Gleason score ≥ 7 compared to Gleason score = 6 are represented by red and blue arrows, respectively

**Fig. 5:**
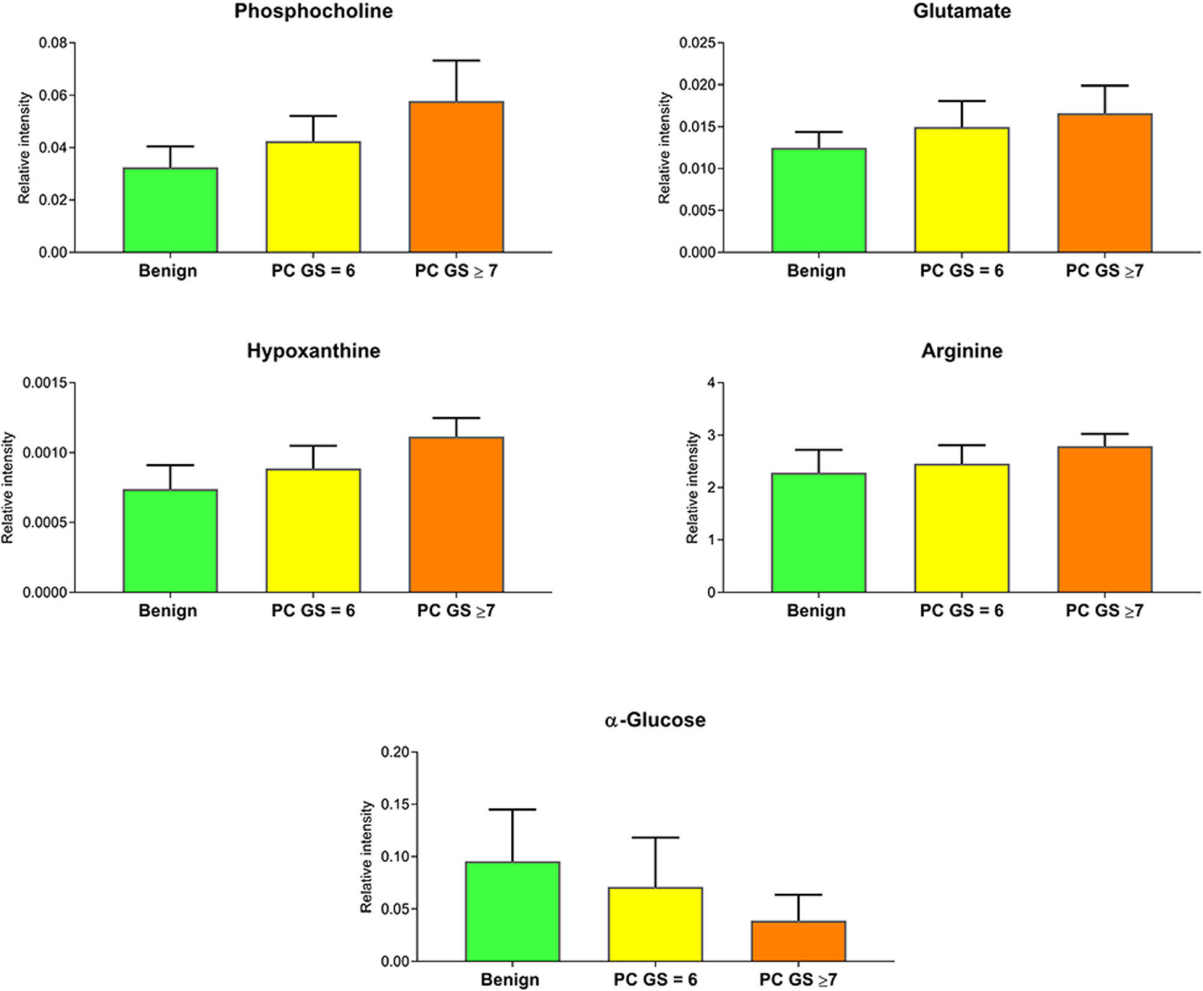
Common significant metabolites discriminating malignant samples from benign samples and high Gleason score compared to low Gleason score. Box and whisker plots illustrating normalized intensities differences between benign samples (green box), PC Gleason score = 6 (yellow box) and PC Gleason score ≥ 7 (orange box)

Metabolomic alterations were found to be specific for PC compared to benign tissues as well as for high-score tumors in relation to low-score tumors; with the most significant alterations being summarized in Fig. 4. The specific aim of the work here was to identify metabolomic changes which unambiguously separate PC from benign samples, and also indicate the progressive changes occurring from low-score to high-score tumors. Therefore, metabolites were compared by applying two group analysis and scrutinized following the pattern of increment or decrement from the benign state to GS = 6 and next to GS ≥ 7. This way, five key metabolites could be identified with all of them following the pattern of PC disease progression (see Fig. 5). In the case of the metabolites phosphocholine, glutamate, hypoxanthine and arginine an increase was observed with progression while α-glucose levels showed a steady decrease.

### Discrimination between ERG-positive and ERG-negative PC

**Fig. 6:**
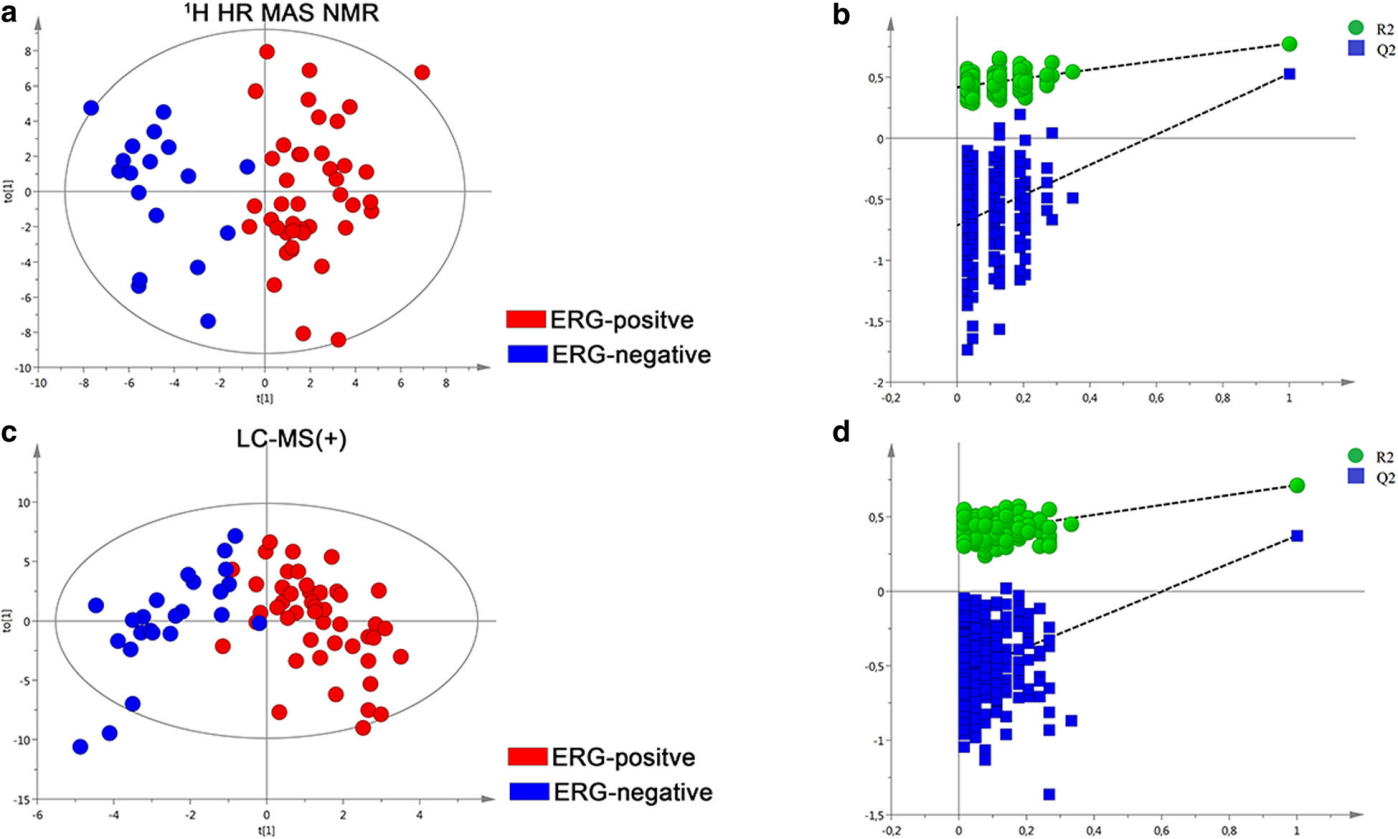
Tissue metabolomics multivariate analysis of ERG-positive PC and ERG-negative PC. **a**. OPLS-DA score plots of ERG-negative samples (bleu dots) and ERG-positive samples (red dots) of ^1^H HR MAS NMR data, **b**. Plot obtained after performing a random permutation test with 200 permutations on OPLS-DA model of ^1^H HR MAS NMR data, **c**. OPLS-DA score plots of ERG-negative samples (bleu dots) and ERG-positive samples (red dots) of LC-MS (+) data, **d**. Plot obtained after performing a random permutation test with 200 permutations on OPLS-DA model of LC-MS (+) data

Data from all five platforms were examined by multivariate analysis in order to create an overview of the metabolic variation in PC tissue samples related to the *TMPRSS2-ERG* gene fusion and related protein expression. The resulting multivariate OPLS-DA classification models revealed a clear separation between predefined ERG-positive and ERG-negative PC samples based on their metabolic profiles from ^1^H HR MAS NMR and LC-MS (+) analysis (see Fig. 6a, c). The models were evaluated using significance testing by means of ANOVA of the cross-validated model with all values summarized in Additional file 3: Table S2. The obtained values indicated that the models were highly significant. Furthermore, a permutation test confirmed the robustness of both OPLS-DA models in distinguishing between ERG-positive and –negative PC tissue samples (Fig. 6b – ^1^H HR MAS NMR-based model and Fig. 6d – LC-MS (+)-based model). Analysis of the model loading plots followed by statistical analyses indicated that twenty-five metabolites contributed to the discrimination between groups (Table 4). Principal component analysis score plots for each data set are shown in Additional file 7: Figure S5A-E.

**Table 4:** Metabolic alterations in ERG Rearrangement-positive PC versus ERG Rearrangement-negative PC

Metabolite	Change in ERG-positive PC	Technique	BH *p*-value^a^	BH *p*-value for mixed model	BH *p*-value for mixed model adjusted for Gleason score	BH *p*-value for mixed model adjusted for percentage of epithelium	BH *p*-value for mixed model adjusted for percentage of malignancy
Glycerophosphocholine	↓	^1^H HR MAS NMR	7.05 × 10^−3^	4.50 × 10^−01^	7.69 × 10^− 01^	4.03 × 10^− 01^	3.79 × 10^− 01^
O-Phosphocholine	↓	^1^H HR MAS NMR	2.96 × 10^− 2^	5.43 × 10^− 01^	7.47 × 10^− 01^	5.31 × 10^− 01^	5.47 × 10^− 01^
Lysine	↓	^1^H HR MAS NMR	1.64 × 10^− 2^	8.85 × 10^− 02^	6.46 × 10^− 01^	1.05 × 10^− 02^	2.00 × 10^− 02^
Tyrosine	↓	^1^H HR MAS NMR	8.74 × 10^− 3^	1.54 × 10^− 03^	2.40 × 10^− 01^	1.54 × 10^− 03^	2.81 × 10^− 03^
Myo-inositol	↑	^1^H HR MAS NMR	4.73 × 10^−3^	3.00 × 10^− 02^	2.99 × 10^− 01^	5.09 × 10^− 02^	5.82 × 10^− 02^
Valine	↓	^1^H HR MAS NMR	1.81 × 10^− 2^	6.91 × 10^− 02^	6.46 × 10^− 01^	3.09 × 10^− 02^	3.54 × 10^− 02^
Phenylalanine	↓	^1^H HR MAS NMR	3.52 × 10^− 2^	1.34 × 10^− 02^	2.89 × 10^− 01^	1.39 × 10^− 02^	2.00 × 10^− 02^
Hypoxanthine	↓	^1^H HR MAS NMR	1.71 × 10^− 2^	8.23 × 10^− 02^	6.13 × 10^− 01^	7.59 × 10^− 02^	4.68 × 10^− 02^
Ascorbate	↓	^1^H HR MAS NMR	3.57 × 10^− 2^	3.20 × 10^− 01^	7.69 × 10^− 01^	4.03 × 10^− 01^	2.81 × 10^− 01^
Glutathione	↓	^1^H HR MAS NMR	3.57 × 10^− 2^	3.66 × 10^− 01^	7.95 × 10^− 01^	4.93 × 10^− 01^	4.03 × 10^− 01^
Aspartate	↓	^1^H HR MAS NMR	4.15 × 10^− 2^	2.74 × 10^− 01^	7.69 × 10^− 01^	2.92 × 10^− 01^	4.28 × 10^− 01^
Butyrylcarnitine	↑	LC-MS (+)	6.90 × 10^− 4^	1.63 × 10^− 01^	3.46 × 10^− 01^	1.57 × 10^− 01^	1.57 × 10^− 01^
Myristoylcarnitine	↑	LC-MS (+)	3.06 × 10^− 4^	8.85 × 10^− 02^	1.88 × 10^− 01^	5.99 × 10^− 02^	3.76 × 10^− 02^
Hexanoylcarnitine	↑	LC-MS (+)	6.90 × 10^− 4^	2.30 × 10^− 01^	6.08 × 10^− 01^	2.50 × 10^− 01^	2.42 × 10^− 01^
Xanthine	↑	LC-MS (+)	2.84 × 10^−4^	8.97 × 10^− 04^	3.42 × 10^− 03^	1.54 × 10^− 03^	2.81 × 10^− 03^
Acetylcarnitine	↑	LC-MS (+)	3.46 × 10^− 4^	9.15 × 10^− 01^	3.31 × 10^− 01^	9.77 × 10^− 02^	1.12 × 10^− 01^
Adenine	↑	LC-MS (+)	1.55 × 10^− 3^	1.75 × 10^− 01^	2.89 × 10^− 01^	1.69 × 10^− 01^	1.57 × 10^− 01^
Palmitoylcarnitine	↑	LC-MS (+)	2.76 × 10^− 3^	1.78 × 10^− 01^	2.89 × 10^− 01^	1.26 × 10^− 01^	1.27 × 10^− 01^
Sphingosine	↓	LC-MS (+)	8.34 × 10^− 3^	2.75 × 10^− 01^	6.13 × 10^− 01^	3.32 × 10^− 01^	3.08 × 10^− 01^
Dodecanoylcarnitine	↑	LC-MS (+)	2.58 × 10^− 3^	2.67 × 10^− 01^	4.73 × 10^− 01^	2.92 × 10^− 01^	2.34 × 10^− 01^
Oleoylcarnitine	↑	LC-MS (+)	1.70 × 10^− 2^	4.77 × 10^− 01^	5.60 × 10^− 01^	3.61 × 10^− 01^	3.36 × 10^− 01^
Stearoylcarnitine	↑	LC-MS (+)	4.26 × 10^− 3^	1.81 × 10^− 01^	2.89 × 10^− 01^	1.25 × 10^− 01^	1.27 × 10^− 01^
Inosine	↑	LC-MS (+)	8.44 × 10^− 4^	1.78 × 10^− 01^	4.73 × 10^− 01^	1.80 × 10^− 01^	1.89 × 10^− 01^
Propionylcarnitine	↑	LC-MS (+)	2.90 × 10^−2^	4.05 × 10^− 01^	7.35 × 10^− 01^	4.22 × 10^− 01^	4.03 × 10^− 01^
Uric acid	↑	LC-MS (+)	2.16 × 10^− 3^	1.75 × 10^− 01^	4.73 × 10^− 01^	1.57 × 10^− 01^	1.83 × 10^− 01^

*BH* Benjamini–Hochberg multiple testing correction; ^a^ nonparametric *t*-test

**Fig. 7:**
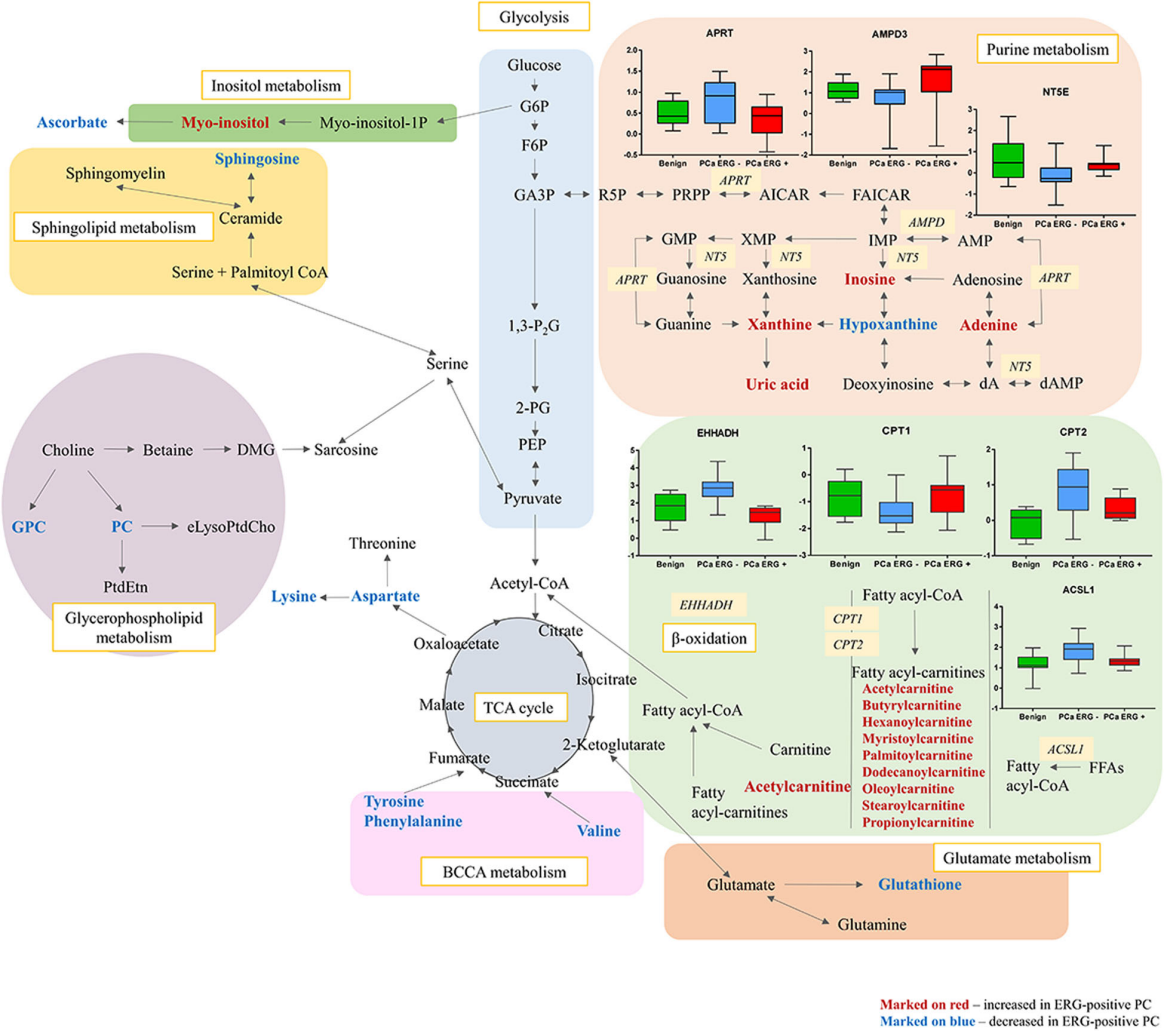
Combined proteomics and metabolomics pathway network map of significantly altered metabolites and proteins in ERG-positive prostate cancer compared to ERG-negative prostate cancer. Metabolites significantly increased in ERG-positive PC are marked on red, significantly decreased in ERG-positive PC are marked on blue. Significantly altered proteins are presented on box and whisker plots illustrating normalized intensities differences between benign samples (green box), ERG-negative PC (blue box) and ERG-positive PC (red box)

The results indicated different metabolic processes in ERG-positive compared to ERG-negative PC, as presented in Fig. 7 as a metabolic map. Decreased levels of sphingosine pointed to a dysregulation of the sphingolipid pathway. Furthermore, different levels of glycerophosphocholine, phosphocholine and myo-inositol pointed towards disturbances in choline metabolism. In addition, the levels of many amino acids were significantly lower in ERG-positive than in -negative PC samples. Interestingly, ERG-positive PC showed increased levels of several acylcarnitines, suggesting a disturbed fatty acid metabolism. Finally, increased levels of metabolites belonging to the purine catabolism reflected presumably a homeostatic response to oxidative stress.

### Proteomic analysis of ERG-positive and ERG-negative PC tissue versus benign prostate tissue

The obtained metabolomic data strongly indicate, that *TMPRSS2-ERG* rearrangement in PC is related to changes in β-oxidation and purine metabolism. To provide further evidence for a mechanistic link to ERG expression, we investigated an existing proteomic data set of non-malignant and malignant tissue samples from 28 radical prostatectomy patients [23] from separate cohort. Focus was on the differences between ERG-positive (*n* = 12) and ERG-negative (*n* = 16) samples, especially on levels of proteins involved the β-oxidation and purine metabolism pathways. As shown in Fig. 7, ERG-positive prostate tumors indicated decreased levels of some proteins involved in mitochondrial β-oxidation; carnitine palmitoyltransferase 2 (CPT2) (*p* = 0.018), peroxisomal protein enoyl-CoA (EHHADH) (*p* < 0.0001) and long-chain-fatty-acid-CoA ligase 1 (ACSL1) (*p* = 0.215), but increased levels of carnitine palmitoyltransferase 1A (CPT1) (*p* = 0.021) in comparison to ERG-negative prostate tumors. Surprisingly, ACSL1, CPT1, CPT2, and EHHADH protein levels found in ERG-positive PC, were similar to the levels found in benign neighboring tissue. However, in ERG-negative PC relatively higher levels were detected compared to benign prostate tissue (Fig. 7). Moreover, for key proteins involved in the purine pathway, pronounced differences in their relative concentrations were found depending on tissue type. In contrast to ERG-negative samples, ERG-positive tissues displayed a decreased level of adenine phosphoribosyltransferase (APRT) (*p* = 0.018), while the concentration of adenosine monophosphate deaminase 3 (AMPD3) (*p* = 0.048) and 5′-Nucleotidase Ecto (NT5E) (*p* = 0.024) was increased (Fig. 7). Like in the β-oxidation pathway, proteins belonging to the purine pathway showed similar levels in the ERG-positive group as in benign tissue. All detected proteins in purine and β-oxidation pathways are listed in Additional file 8: Table S3.

## Discussion

In this study, we established an approach for integrating information originating from distinct analytical methods, to generate tissue specific metabolite profiles, which showed characteristic and unambiguous alterations in PC, high-score PC, and *TMPRSS2*-ERG-positive PC, respectively. To achieve this accuracy, the workflow developed here (see Fig. 1) enabled histological examinations in parallel to the comprehensive metabolomic analysis of each tissue sample by various analytical approaches, ranging from, ^1^H HR MAS NMR on the intact tissues to liquid ^1^H NMR, ^31^P NMR and LC-MS analysis of the corresponding tissue extracts. We used a mild extraction protocol without any requirement for tissue homogenization [19]; an approach which left the morphology and structure of the tissue specimen intact and allowed thorough histopathological analysis of the same specimen. Since deuterated water (for intact tissue ^1^H HR MAS NMR required as NMR ^2^H spin-lock signal) and deuterated solvents for extraction were used, extracts could be analyzed by liquid NMR and LC-MS without any additional (potentially damaging) steps of solvent exchange [25]. In total, 136 metabolites were identified using all four analytical platforms together. Many of these metabolites were detected simultaneously by different platforms. Nevertheless, the main focus was on the verification of our integrated approach rather than obtaining the highest number of identities. As shown here, the complementary nature of these four different techniques offers an insightful approach to understand the differences in the metabolic profiles of PC, in the context of GS and *TMPRSS2-ERG*-fusion status.

### Metabolism in PC

Five key metabolites (phosphocholine, glutamate, hypoxanthine, arginine, α-glucose) emerged here, whose appearance and deviations in relative levels allowed an unambiguous differentiation between cancer and benign tissue and even between high and low GS. Patterns observed for these metabolites also reflected the progressive changes occurring from benign to low-score PC and then to high-score PC (Fig. 5). Therefore, these five metabolites could be highly indicative markers for tumor progression and disease aggressiveness.

Glucose levels were reduced in PC tissues, as seen in other cancers [26–28]. The correlation between diminishing glucose concentrations with increasing GS pinpoints glycolysis as preferred pathway for generating the metabolic intermediates needed for de novo biosynthesis to support cell proliferation. Besides glycolysis, increased glutaminolysis is recognized as a vital metabolism pathway of cancer cells to meet the high-energy demand under hypoxic conditions [29]. For glutamate increased levels were seen in PC, and these levels were positively correlated with a higher GS. Another hallmark of cancer cells is an intensified de novo lipogenic signature reflecting the need of an increased lipid generation for cell proliferation [30]*.* Phospholipids are playing a vital active role in cellular physiology by mediating key signal transduction pathways controlling cellular survival and proliferation [20]. Higher levels of the lipid phosphocholine were observed in PC compared to normal prostate; as seen even in other malignant tumors [26, 31]*.* Additionally, significant differences were seen in phosphocholine levels between high-score and low-score PC. Already previous ex vivo studies indicated correlations between GS and choline metabolism [32, 33]. The most significant metabolic perturbations visible between the five key metabolites, were the severely increased levels found for arginine and hypoxanthine. Arginine and its products are critical for tumor growth of several cancers, and arginine depletion has been shown to be effective as anti-cancer therapy including even PC ones [34, 35]. Increase in hypoxanthine reflects most likely an upregulation in purine metabolism due to hypoxia and oxidative stress, with both occurring during PC development [36]. Further metabolic changes were observed which were either specific for discriminating PC from benign samples (see Table 2) or between high and low GS (see Table 3).

We also validated our results by comparison with the metabolomics alterations on PC tissues found in previous studies [37, 38]. Sreekumar et al. [39] reported a significant increase of six metabolites including sarcosine, uracil, kynurenine, glycerol-3-phosphate, leucine and proline, during disease progression from benign to PC to metastatic samples. Those results were confirmed by McDunn et al. [40], who also found metabolites like proline, malate, ADP-ribose and 6-sialyl-N-acetyllactosamine being mostly associated with Gleason pattern progression. Like in those two studies we observed an increase in levels of uracil in PC. Another interesting approach of metabolomic profiling of intact tissue was presented by Huan et al. [24]; an approach based on molecular preservation by extraction and fixation and high-performance chemical isotope labeling LC-MS. They proposed a subset of five metabolites, including, adenosine monophosphate, uracil and spermidine, significant in comparison between PC and normal samples. Uracil was again a common metabolite as also found by us here, and additionally spermidine, belonging to the group of polyamines. Our observed changes in the levels of polyamines were also confirmed by an previous study by Huang et al. [41]. As reported by Jung et al. [42], we also observed increased levels in fatty acids in PC and again an increase in choline-containing metabolites. There are some variations in the results reported by these previous studies and our findings. One reason could be that different extraction methods were used; with our method being milder to allow subsequent histopathology upon NMR measurements of intact specimens. Another reason could be that – in contrast to us – many other studies did not correlate metabolomic profile outcome with exact histopathological analysis and could therefore not correct for important factors, like tumor load and grade. Interestingly, our metabolomics profile of cancer samples share common pattern of changes with another study also using the ^1^H HR MAS NMR technique [43]. These changes include decreased levels of polyamines, citrate and glucose and increased levels of choline-containing compounds, succinate and glutamate. Authors of this study also compared high and low tumor grade and proposed citrate and spermine as a biomarkers of PC aggressiveness. Here, the variations observed by us were not significant enough to recommend citrate and polyamines as metabolic biomarkers for PC aggressiveness. These deviations might be explained in the higher number of samples with GS ≥ 8 in their study [43], while we had only one sample in that range.

### Metabolism in relation to ERG rearrangement

*ERG* is one of the most consistently overexpressed oncogenes in malignant PC and there is increasing evidence that it is crucially implicated in the etiology of PC [7]. Understanding the molecular heterogeneity between *ERG* rearrangement-positive and *ERG* rearrangement-negative PC may unlock novel prognostic and therapeutic biomarkers for PC, a major aim in this study. Prior the work presented here, only two reports showed any influence of ERG on the metabolome. Meller et al. [15] pointed at an altered fatty acid oxidation in ERG-positive tumors and Hansen et al. [14] established a connection between the tissue metabolic profile of *TMPRSS2-ERG* and the metabolism of polyamines and citrate, and also glycolysis and fatty acid metabolism. Their results indicated that *TMPRSS2-ERG* differentiates PC towards an aggressive phenotype. Comparison of ERG-positive and -negative tumors in our study showed significant changes over a wide range of metabolites. Most of them belonged to β-oxidation and purine pathways, a conclusion further validated by external proteomic data originating from a separate cohort of patients.

Here, significantly higher levels of acyl-carnitines in ERG-positive PC were observed as indication of alterations in the β-oxidation metabolism between ERG-positive and -negative PC. Acyl-carnitines have recently gained considerable interest in cancer research [44]. Lu et al. [45] proposed serum acetylcarnitine as a biomarker of hepatocellular carcinoma. Increased level of acylcarnitines have also been associated with development of colorectal tumors [46]. Furthermore, differences in levels of acylcarnitines were seen between subtypes of breast cancer [47]. Several acylcarnitines showed increased levels in the urine of kidney cancer patients and in patients with high cancer grades [48]. Many studies also suggested that alteration in β-oxidation might play an important role in the pathogenesis and progression of PC. These suggestions were further confirmed by proteomics data indicating an upregulation of fatty acid oxidation [23]. Even peroximal branched chain fatty acid β-oxidation was upregulated in PC [49], and lipids were also suggested as potential markers of metastatic PC [50]. Importantly, the results of our study here, suggest that an increase in β-oxidation can be mainly attributed to *TMPRSS2-ERG*-negative tumors, while ERG*-*positive tumors instead accumulate acetylcarnitines, most likely due to reduced levels in proteins involved in mitochondrial β-oxidation.

In ERG-positive PC, disturbances were also detected for metabolites belonging to the purine catabolism pathway, namely elevated levels for inosine, xanthine and uric acid and decreased hypoxanthine levels. Moreover, three proteins (APRT, AMPD3 and NT5E) from this pathway showed significantly enhanced levels in ERG-positive cases of the validation cohort. These changes are indicative for oxidative stress and high tumor cell turnover of nucleotides to nucleosides. Experimental and clinical studies suggest that oxidative stress plays a major role in explaining PC development and progression [51]. Moreover, purines are essential for cell proliferation and their inhibition can lead to apoptosis [52]. Taken together, our results thus indicate that the purine degradation cycle is higher in *TMPRSS2-ERG*-negative tumors.

We found significantly lower levels for many amino acids in ERG rearrangement-positive PC samples, possibly suggesting a particularly high demand of amino acids in this tumor subtype. Also increased levels of sphingosine were detected, indicating that these membrane building sphingolipids play also a significant role in tumorigenesis [53]. PC samples with positive- ERG rearrangement showed also reduced levels of glycero-3-phosphocholine and phosphocholine; an observation indicating presumably an extensive turnover of cell membranes. Even, the decreased level of glutathione found, is an important indicator of oxidative stress in ERG-positive PC, while the observed attenuation of myo-inositol levels in ERG-positive PC could be indicative of a change in PI3K-AKT-mTOR signaling pathway. Activation of this pathway is mainly caused by the common loss of function of phosphatase and tensin homologue (PTEN) in PC [54]. It has been shown that ERG rearrangements and PTEN loss are concurrent events that collaboratively stimulates PC development and progression [55–57]. Therefore, as suggested by Squire [58], future therapies developed for treatment of ERG- positive PC should probably target not only the ETS pathway, but also the PTEN pathway.

## Conclusions

The study presented here identified a group of metabolites that do not only constitute potential biomarkers for aggressive PC, but also provide molecular information about underlying biochemical mechanisms. This information can be useful for design novel diagnostic and therapeutic approaches for further validation in considerably larger patient cohorts. The detected metabolomics-derived markers associated with high GS, could be exploited in magnetic resonance imaging or positron emission tomography (PET) imaging approaches for noninvasive, in vivo detection of clinical relevant PC. Analogues of phosphocholine, glutamate and glucose, as identified here, are already applied in PC studies. The 11C/18F choline-based agents are lipid-metabolism PET tracers that have been approved by the U.S. Food and Drug Administration for PET imaging of recurrent PC. Several 11C- and 18F-labeled glutamine analogs have been used as PET tumor-imaging agents, and 18F-fluorodeoxyglucose PET is an analog of glucose that reflects local rates of glucose consumption by tissues [59, 60]. Furthermore, our results highlight two additional metabolites, hypoxanthine and arginine, being associated with PC occurrence and progression.

The observed metabolic differences between ERG-positive and ERG-negative PC indicate that the increase in β-oxidation and purine metabolism often reported for PC could be mainly attributed to *TMPRSS2-ERG*-negative tumors. Taken together, our results strongly support the view that ERG-positive and ERG-negative PC should be considered as partly different diseases probably requiring different treatment strategies.

## Supplementary information

**Additional file 1: Table S1.** Identified metabolites by ^1^H HR MAS NMR, ^1^H NMR, ^31^P NMR, LC-MS positive (+) and negative (−) mode.

**Additional file 2: Figure S1. A-E** Principal component analysis score plots of benign samples (green dots) and malignant samples (brown dots) **a**. ^1^H HR MAS NMR data, **b**. ^1^H NMR data, **c**. ^31^P NMR data, **d**. LC-MS (+) data, **e**. LC-MS (−) data.

**Additional file 3: Table S2.** Overview of multivariate models.

**Additional file 4: Figure S2. A-E** Plots obtained after performing a random permutation test with 200 permutations on OPLS-DA model of benign samples and malignant samples **A.**^1^H HR MAS NMR data, **B.**^1^H NMR data, **C.**^31^P NMR data, **D.** LC-MS (+) data, **E.** LC-MS (−) data.

**Additional file 5: Figure S3. A-E** Principal component analysis score plots of Gleason score = 6 PC samples (orange dots) and Gleason score ≥ 7 PC samples (red dots) **A.**^1^H HR MAS NMR data, **B.**^1^H NMR data, **C.**^31^P NMR data, **D.** LC-MS (+) data, **E.** LC-MS (−) data.

**Additional file 6: Figure S4. A-E** Plots obtained after performing a random permutation test with 200 permutations on OPLS-DA model of Gleason score = 6 PC samples and Gleason score ≥ 7 PC samples **A.**^1^H HR MAS NMR data, **B.**^1^H NMR data, **C.**^31^P NMR data, **D.** LC-MS (+) data, **E.** LC-MS (−) data.

**Additional file 7: Figure S5. A-E** Principal component analysis score plots of ERG-negative samples (bleu dots) and ERG-positive samples (red dots) **A.**^1^H HR MAS NMR data, **B.**^1^H NMR data, **C.**^31^P NMR data, **D.** LC-MS (+) data, **E.** LC-MS (−) data.

**Additional file 8: Table S3.** Detected proteins in β-oxidation and purine pathways.

## Data Availability

The dataset used and/or analyzed during the current study are available from the corresponding author on reasonable request.

## Abbreviations

ACSL1: Long-chain-fatty-acid-CoA ligase 1
AMPD3: Adenosine monophosphate deaminase 3
APRT: Adenine phosphoribosyltransferase
CPMG: Carr-Purcell- Meiboom-Gill
CPT2: Carnitine palmitoyltransferase 2
CV-ANOVA: Cross-validation-analysis of variance
EHHADH: Peroxisomal protein enoyl-CoA
ERG: Erythroblast transformation-specific (*ETS*) transcriptional factor ETS-related gene
FFPE: Formalin fixed paraffin embedded
GS: Gleason score
H&E: hematoxylin-eosin
^1^H HR MAS NMR: Proton high resolution magic angle spinning nuclear magnetic resonance
^1^H NMR: Proton nuclear magnetic resonance
LC-MS: Liquid chromatography-mass spectrometry
LC-MS (+): Liquid chromatography-mass spectrometry positive mode
LC-MS (−): Liquid chromatography-mass spectrometry negative mode
NT5E: 5′-Nucleotidase Ecto
OCT: Optimal Cutting Temperature
OPLS-DA: Orthogonal partial least squares discriminant analysis
PC: Prostate cancer
PET: Positron emission tomography
^31^P NMR: Phosphorus nuclear magnetic resonance
*TMPRSS2*: Androgen-responsive promotor transmembrane protease, serine 2
