# Supplementary material for: Comprehensive metabolomics analysis of prostate cancer tissue in relation to tumor aggressiveness and TMPRSS2-ERG fusion status
Source: BMC Cancer. 2020 May 18;20:437. doi: 10.1186/s12885-020-06908-z (PMC7236196; doi:10.1186/s12885-020-06908-z)
Supplement: Supplementary file 1 — Additional file 1: Table S1. Identified metabolites by 1H HR MAS NMR, 1H NMR, 31P NMR, LC-MS positive (+) and negative (−) mode. [file 12885_2020_6908_MOESM1_ESM.docx]

**Table S1.** Identified metabolites by ^1^H HR MAS NMR, ^1^H NMR, ^31^P NMR, LC-MS positive (+) and negative (-) mode.

|  | Name | Technique |
| --- | --- | --- |
| 1 | 5'-AMP (Adenosine5'-monophosphate) | LC-MS (+) |
| 2 | 5'-CMP (Cyclic adenosine monophosphate) | LC-MS (+) |
| 3 | 1.5-Anhydrosorbitol | LC-MS (+) |
| 4 | 10Z-Heptadecenoic acid (17:1) | LC-MS (-) |
| 5 | 2-Aminobutyric acid or Aminobutryic acid (GABA) | LC-MS (+), LC-MS (-) |
| 6 | 2-Hydroxybutyrate | ^1^H NMR |
| 7 | 2-Oxoisocaproic acid | LC-MS (-) |
| 8 | 3-hydroxybutarate | ^1^H HR MAS NMR, ^1^H NMR, LC-MS (-) |
| 9 | 3-Methyl-L-histidine | LC-MS (+) |
| 10 | Acetylcarnitine | LC-MS (+) |
| 11 | Adenine | LC-MS (+) |
| 12 | Adenosine | ^1^H NMR, LC-MS (+) |
| 13 | Alanine | ^1^H HR MAS NMR, ^1^H NMR |
| 14 | Arachidic Acid (20:0) | LC-MS (-) |
| 15 | Arginine | ^1^H NMR, LC-MS (+), LC-MS (-) |
| 16 | Ascorbate | ^1^H HR MAS NMR, ^1^H NMR |
| 17 | Asparagine | LC-MS (+) |
| 18 | Aspartate | ^1^H HR MAS NMR, ^1^H NMR, LC-MS (+) |
| 19 | Benzoic acid | LC-MS (-) |
| 20 | Butyryl-L-carnitine | LC-MS (+) |
| 21 | Caffeine | LC-MS (+) |
| 22 | Cardiolipin | ^31^P NMR |
| 23 | Carnitine | LC-MS (+) |
| 24 | Ceramide-1-phospate | ^31^P NMR |
| 25 | Chenodeoxycholic acid glycine conjugate | LC-MS (-) |
| 26 | Choline | ^1^H HR MAS NMR |
| 27 | Choline phosphate | ^31^P NMR |
| 28 | cis-9-Palmitoleic acid (16:1) | LC-MS (-) |
| 29 | cis-Gondoic acid (20:1) | LC-MS (-) |
| 30 | Citrate | ^1^H HR MAS NMR, ^1^H NMR, LC-MS (+), LC-MS (-) |
| 31 | Citrulline | LC-MS (-) |
| 32 | Creatine | ^1^H HR MAS NMR, ^1^H NMR, LC-MS (+), LC-MS (-) |
| 33 | Creatinine | LC-MS (+), LC-MS (-) |
| 34 | Dihydroceramide-1-phospate | ^31^P NMR |
| 35 | Docosahexaenoic acid (22:6) | LC-MS (-) |
| 36 | Docosapentaenoic acid (DPA) (22:5) | LC-MS (-) |
| 37 | Dodecanoylcarnitine | LC-MS (+) |
| 38 | Ethanolamine phosphate | ^31^P NMR |
| 39 | Fructose | LC-MS (-) |
| 40 | Fumarate | ^1^H HR MAS NMR, ^1^H NMR |
| 41 | Glucose 6-phosphate or Glucose 1-phosphate | LC-MS (+) |
| 42 | Glutamate | ^1^H HR MAS NMR, ^1^H NMR, LC-MS (+) |
| 43 | Glutamine | ^1^H HR MAS NMR, ^1^H NMR, LC-MS (+) |
| 44 | Glutaric acid (C5-DC) | LC-MS (-) |
| 45 | Glutaroyl carnitine | LC-MS (+) |
| 46 | Glutathione | ^1^H HR MAS NMR |
| 47 | Glutathione oxidized | LC-MS (+) |
| 48 | Glycerate 3-phosphate | LC-MS (+) |
| 49 | Glyceric acid | LC-MS (+) |
| 50 | Glycerophosphocholine | ^1^H HR MAS NMR, ^1^H NMR |
| 51 | GMP (guanosine monophosphate) | LC-MS (+) |
| 52 | Hexanoylcarnitine | LC-MS (+) |
| 53 | Histidine | ^1^H HR MAS NMR |
| 54 | Histidine | LC-MS (-) |
| 55 | Hypoxanthine | ^1^H HR MAS NMR, ^1^H NMR, LC-MS (+), LC-MS (-) |
| 56 | Inosine | ^1^H HR MAS NMR, ^1^H NMR LC-MS (+) |
| 57 | Isoleucine | ^1^H HR MAS NMR, ^1^H NMR, LC-MS (-) |
| 58 | Isovaleryl-carnitine | LC-MS (+) |
| 59 | Kynurenine | LC-MS (+) |
| 60 | Lactet | ^1^H HR MAS NMR, ^1^H NMR |
| 61 | Lauric acid (12:0) | LC-MS (-) |
| 62 | Leucine | 1H HR MAS NMR, 1H NMR, LC-MS (+) |
| 63 | Linoleic acid (18:2) | LC-MS (-) |
| 64 | Lipid (n) CH2 | ^1^H NMR |
| 65 | Lipids (CH2)n | ^1^HR MAS NMR, ^1^H NMR |
| 66 | Lipids (CH2)n (mainly LDL/VLDL) | ^1^H HR MAS NMR, ^1^H NMR |
| 67 | Lipids (CH2-C=C) | ^1^H HR MAS NMR, ^1^H NMR |
| 68 | Lipids (-CH3) (mainly LDL/VLDL) | ^1^H HR MAS NMR, ^1^H NMR |
| 69 | Lipids −CH2−CH2−C=O | ^1^H NMR |
| 70 | Lysine | ^1^H HR MAS NMR, LC-MS (+), LC-MS (-) |
| 71 | Lysophosphatidylcholine | ^31^P NMR |
| 72 | Malate | ^1^H HR MAS NMR, ^1^H NMR |
| 73 | Maleic acid (C4:1-DC) | LC-MS (-) |
| 74 | Maltose | LC-MS (+) |
| 75 | Margaric acid (17:0) | LC-MS (-) |
| 76 | Methionine | ^1^H HR MAS NMR |
| 77 | Methionine | LC-MS (+) |
| 78 | Myo-inositol | ^1^H HR MAS NMR |
| 79 | Myristic acid (14:0) | LC-MS (-) |
| 80 | Myristoleic acid (14:1) | LC-MS (-) |
| 81 | Myristoyl-carnitine | LC-MS (+) |
| 82 | NAD+ | ^1^H NMR |
| 83 | Niacinamide | LC-MS (+) |
| 84 | Oleic acid (18:1) | LC-MS (-) |
| 85 | Oleoyl-carnitine | LC-MS (+) |
| 86 | O-Phosphorylethanolamine | LC-MS (+) |
| 87 | Ornithine | LC-MS (+) |
| 88 | Palmitic acid (16:0) | LC-MS (-) |
| 89 | Palmitoyl-carnitine | LC-MS (+) |
| 90 | Pantothenic Acid | LC-MS (-) |
| 91 | Paraxanthine | LC-MS (+) |
| 92 | PC(16:0/0:0) palmitoyl | LC-MS (+) |
| 93 | PC(18:0/0:0) stearoyl | LC-MS (+) |
| 94 | PC(18:1/0:0) oleoyl | LC-MS (+) |
| 95 | Phenylalanine | ^1^H HR MAS NMR, ^1^H NMR, LC-MS (+), LC-MS (-) |
| 96 | Phosphatidylcholine | ^31^P NMR |
| 97 | Phosphatidylcholine ether | ^31^P NMR |
| 98 | Phosphatidylethanolamine | ^31^P NMR |
| 99 | Phosphatidylethanolamine plasmogen | ^31^P NMR |
| 100 | Phosphatidylglycerol | ^31^P NMR |
| 101 | Phosphatidylinositol | ^31^P NMR |
| 102 | Phosphocholine | ^1^H HR MAS NMR, ^1^H NMR |
| 103 | Pipecolic acid | LC-MS (+) |
| 104 | Polyamines | ^1^H HR MAS NMR, ^1^H NMR |
| 105 | Proline | LC-MS (+), LC-MS (-) |
| 106 | Propionyl-carnitine | LC-MS (+) |
| 107 | Pyroglutamic acid (5-oxoproline) | LC-MS (+) |
| 108 | Ribose | LC-MS (+) |
| 109 | S-Adenosylhomocysteine | LC-MS (+) |
| 110 | Scyllo-inositol | ^1^H HR MAS NMR |
| 111 | Sebacic acid (C10:0-DC) | LC-MS (-) |
| 112 | Serine | ^1^H HR MAS NMR, LC-MS (+), LC-MS (-) |
| 113 | Sorbitol | LC-MS (-) |
| 114 | Spermidine | LC-MS (+) |
| 115 | Sphingosine | LC-MS (+) |
| 116 | Sphingosine-1-phosphate | ^31^P NMR |
| 117 | Sphinomyelin+Phosphatidylserine | ^31^P NMR |
| 118 | Stearic acid (18:0) | LC-MS (-) |
| 119 | Stearoyl-carnitine | LC-MS (+) |
| 120 | Succinate | ^1^H HR MAS NMR, ^1^H NMR, LC-MS (+), LC-MS (-) |
| 121 | Sucrose | LC-MS (-) |
| 122 | Taurine | ^1^H HR MAS NMR, LC-MS (+), LC-MS (-) |
| 123 | Tetradecanedioic acid (C14:0-DC) | LC-MS (-) |
| 124 | Theobromine | LC-MS (+) |
| 125 | Threonine | ^1^H HR MAS NMR, ^1^H NMR, LC-MS (+) |
| 126 | trans-Aconitate | LC-MS (-) |
| 127 | Tryptophan | LC-MS (+) |
| 128 | Tyrosine | ^1^H HR MAS NMR, ^1^H NMR, LC-MS (+), LC-MS (-) |
| 129 | Unsaturated lipids -CH=CH- | ^1^H HR MAS NMR, ^1^H NMR |
| 130 | Uracil | ^1^H HR MAS NMR, LC-MS (+) |
| 131 | Uric acid | LC-MS (+) |
| 132 | Uridine | ^1^H HR MAS NMR, ^1^H NMR |
| 133 | Valine | ^1^H HR MAS NMR, ^1^H NMR, LC-MS (+), LC-MS (-) |
| 134 | Xanthine | LC-MS (+) |
| 135 | α-Glucose | ^1^H HR MAS NMR, ^1^H NMR |
| 136 | α-Linolenic Acid (18:3) | LC-MS (-) |
