# Supplementary figures and images for: Comprehensive metabolomics analysis of prostate cancer tissue in relation to tumor aggressiveness and TMPRSS2-ERG fusion status
Source: BMC Cancer. 2020 May 18;20:437. doi: 10.1186/s12885-020-06908-z (PMC7236196; doi:10.1186/s12885-020-06908-z)

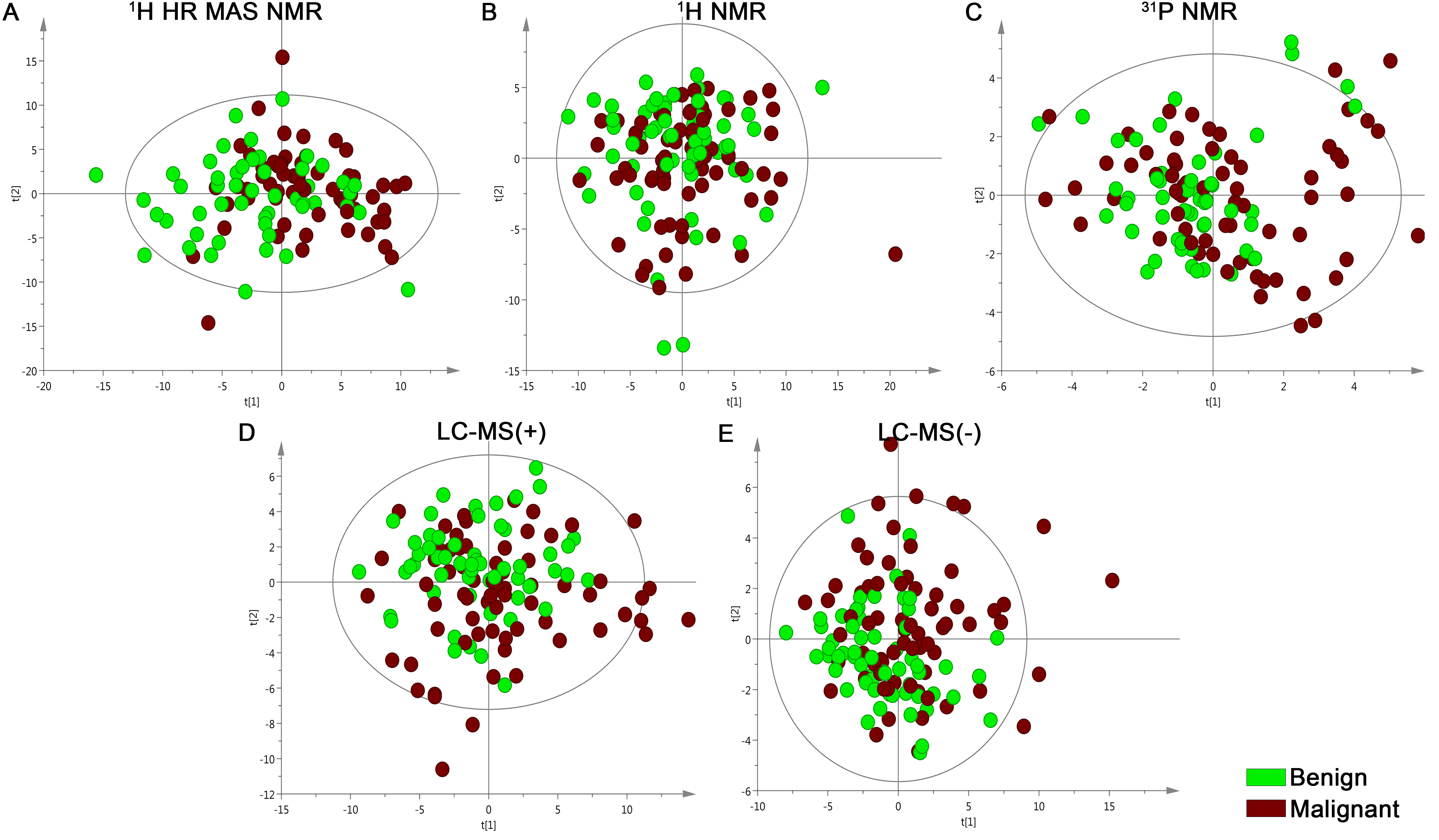

Supplement: Supplementary file 2 — Additional file 2: Figure S1. A-E Principal component analysis score plots of benign samples (green dots) and malignant samples (brown dots) a. 1H HR MAS NMR data, b. 1H NMR data, c. 31P NMR data, d. LC-MS (+) data, e. LC-MS (−) data. [file 12885_2020_6908_MOESM2_ESM.tif]

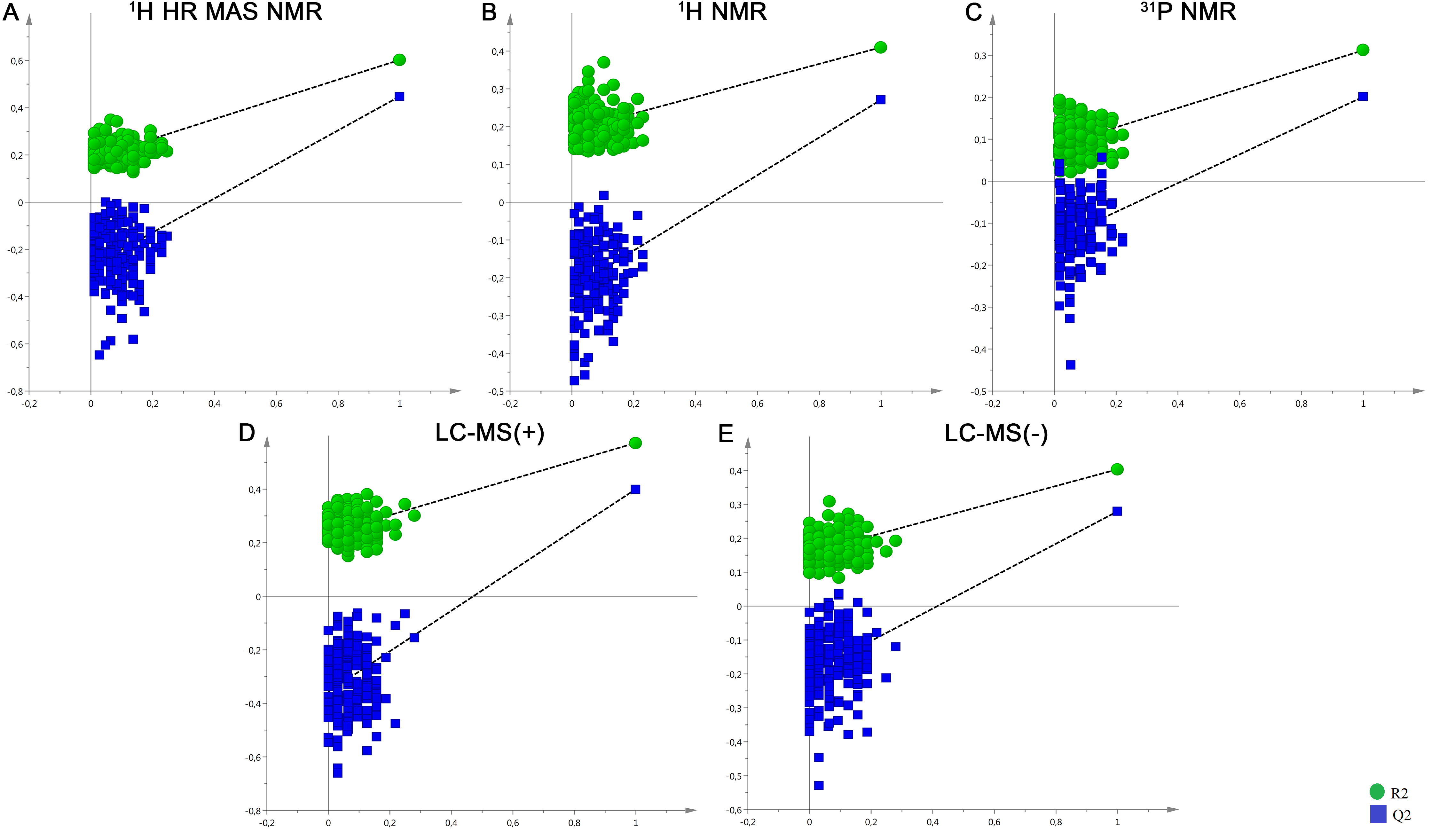

Supplement: Supplementary file 4 — Additional file 4: Figure S2. A-E Plots obtained after performing a random permutation test with 200 permutations on OPLS-DA model of benign samples and malignant samples A.1H HR MAS NMR data, B.1H NMR data, C.31P NMR data, D. LC-MS (+) data, E. LC-MS (−) data. [file 12885_2020_6908_MOESM4_ESM.tif]

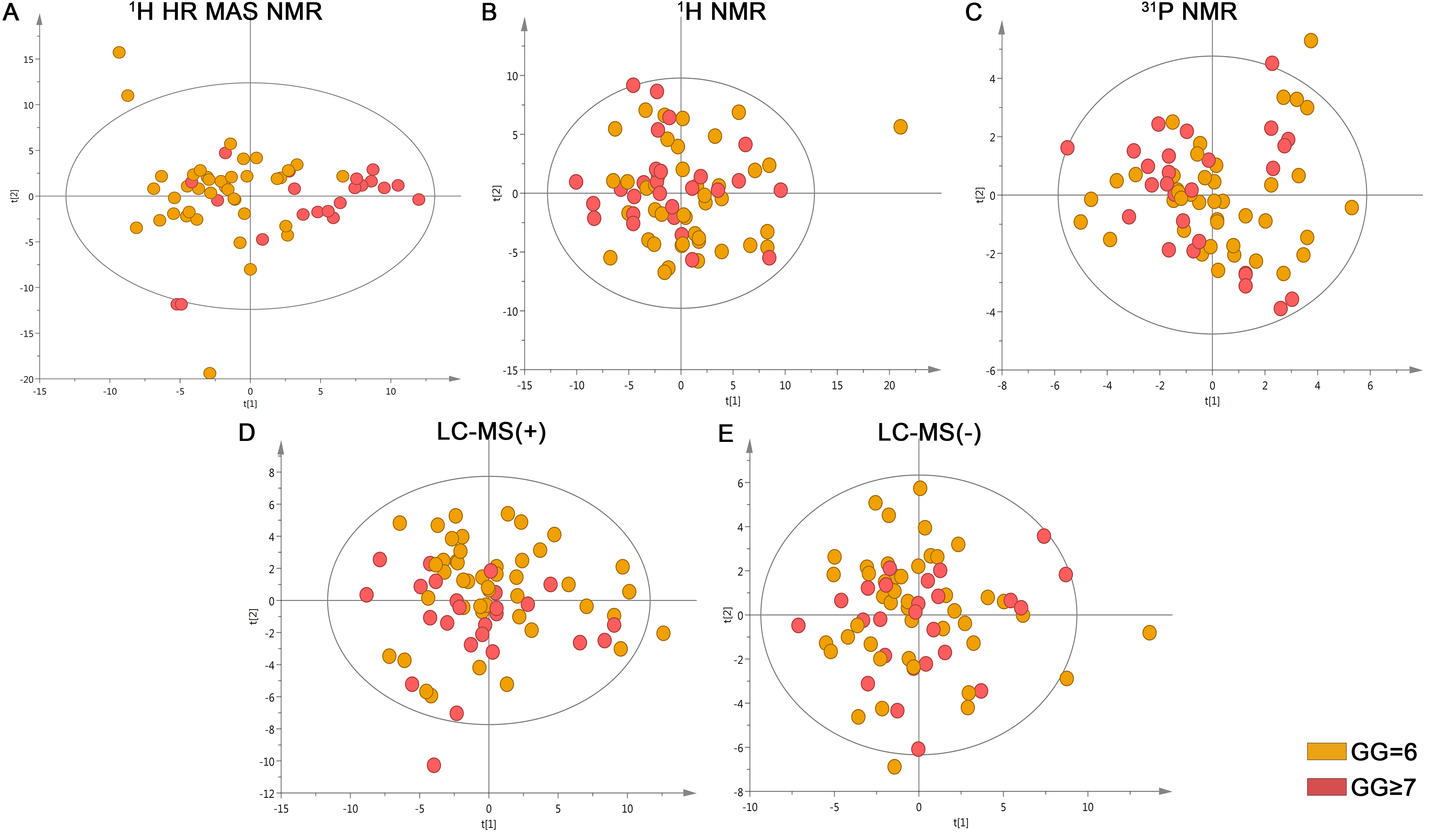

Supplement: Supplementary file 5 — Additional file 5: Figure S3. A-E Principal component analysis score plots of Gleason score = 6 PC samples (orange dots) and Gleason score ≥ 7 PC samples (red dots) A.1H HR MAS NMR data, B.1H NMR data, C.31P NMR data, D. LC-MS (+) data, E. LC-MS (−) data. [file 12885_2020_6908_MOESM5_ESM.tif]

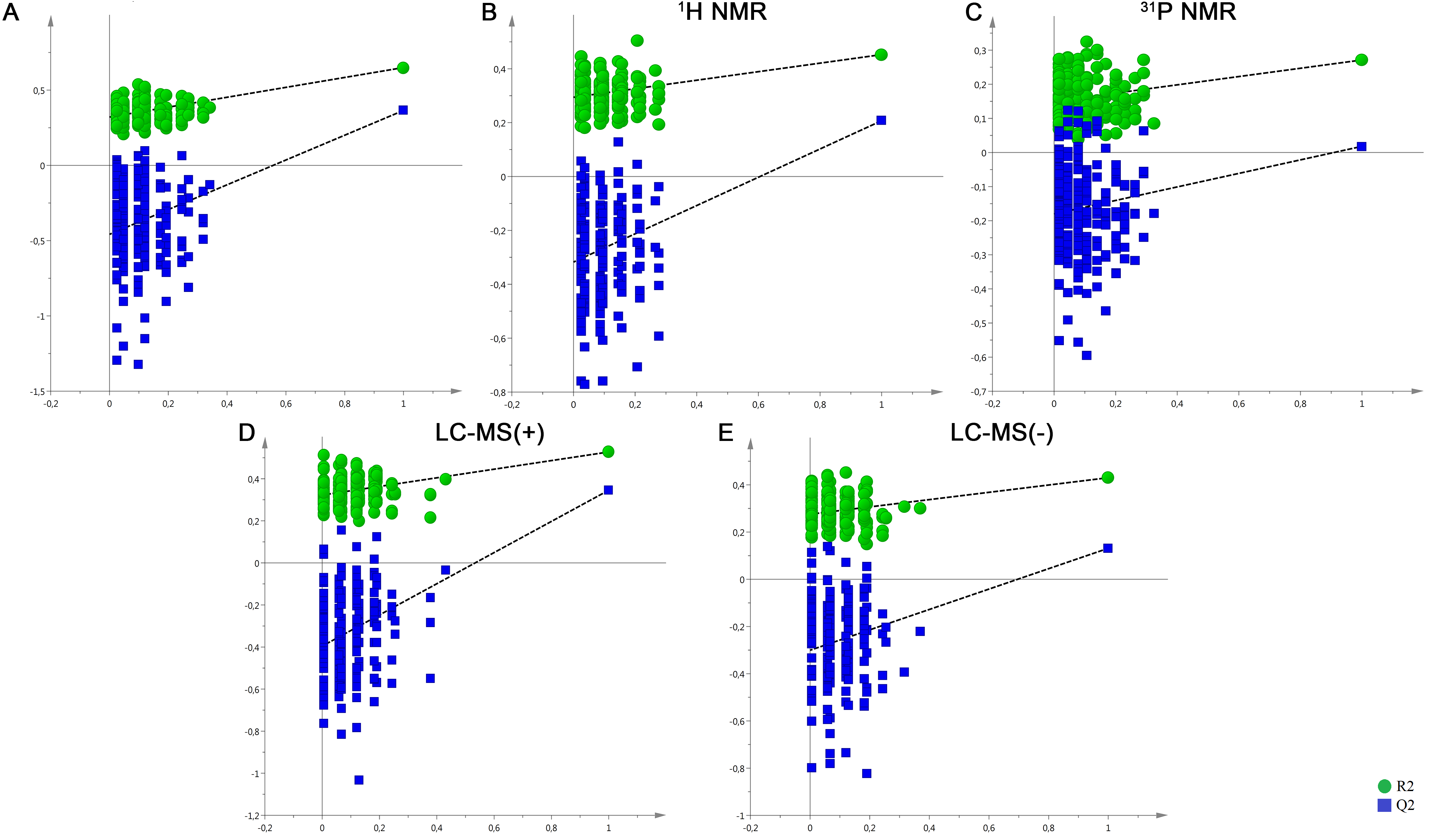

Supplement: Supplementary file 6 — Additional file 6: Figure S4. A-E Plots obtained after performing a random permutation test with 200 permutations on OPLS-DA model of Gleason score = 6 PC samples and Gleason score ≥ 7 PC samples A.1H HR MAS NMR data, B.1H NMR data, C.31P NMR data, D. LC-MS (+) data, E. LC-MS (−) data. [file 12885_2020_6908_MOESM6_ESM.tif]

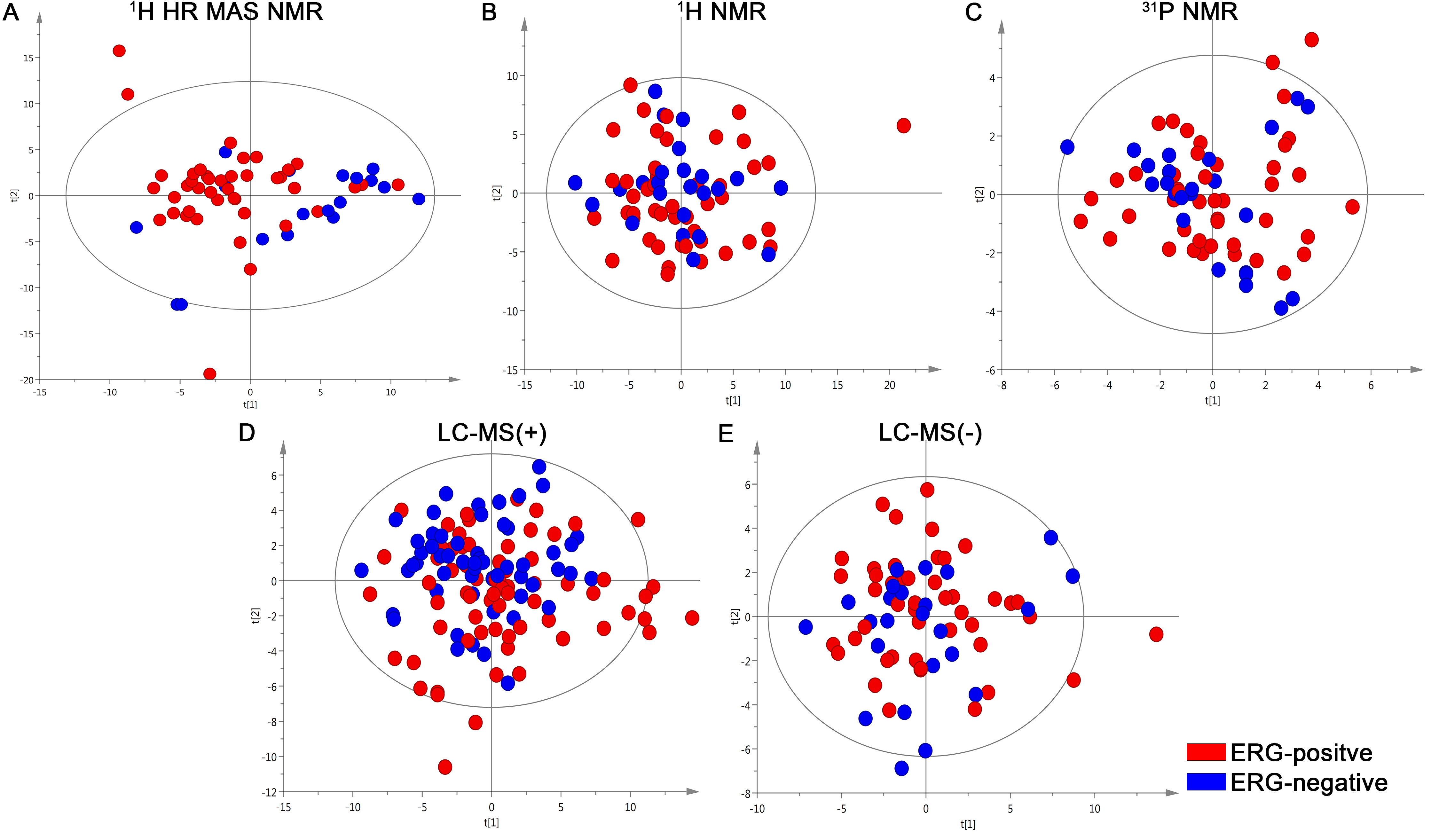

Supplement: Supplementary file 7 — Additional file 7: Figure S5. A-E Principal component analysis score plots of ERG-negative samples (bleu dots) and ERG-positive samples (red dots) A.1H HR MAS NMR data, B.1H NMR data, C.31P NMR data, D. LC-MS (+) data, E. LC-MS (−) data. [file 12885_2020_6908_MOESM7_ESM.tif]
