# Supplementary material for: Comprehensive metabolomics analysis of prostate cancer tissue in relation to tumor aggressiveness and TMPRSS2-ERG fusion status
Source: BMC Cancer. 2020 May 18;20:437. doi: 10.1186/s12885-020-06908-z (PMC7236196; doi:10.1186/s12885-020-06908-z)
Supplement: Supplementary file 3 — Additional file 3: Table S2. Overview of multivariate models. [file 12885_2020_6908_MOESM3_ESM.docx]

**Table S2. Overview of multivariate models.**

**ANALYSIS^a^ MODEL COMP.^b^ N samples^c^ N variables^d^ R^2^X (CUM)^e^ R^2^Y (CUM) ^e^ Q^2^ (CUM) ^e^ CV-ANOVA^f^**

**Comparison of malignant tissue samples with benign samples.**

^1^H HR MAS NMR OPLS-DA 1+1 110 174 0.228 0.603 0.448 6.70 × 10^−13^

^1^H NMR OPLS-DA 1+1 128 103 0.297 0.410 0.271 6.35 × 10^−8^

^31^P NMR OPLS-DA 1+1 120 16 0.364 0.312 0.201 3.10 × 10^−5^

LC-MS (+) OPLS-DA 1+3 129 66 0.548 0.573 0.400 1.30 × 10^−10^

LC-MS (-) OPLS-DA 1+1 129 54 0.322 0.403 0.297 2.74 × 10^−8^

**Comparison of PC samples with high Gleason score (GS ≥ 7) to low Gleason score (GS = 6).**

^1^H HR MAS NMR OPLS-DA 1+1 60 174 0.203 0.65 0.365 4.16 × 10^−5^

^1^H NMR OPLS-DA 1+1 70 103 0.321 0.453 0.207 4.08 × 10^−3^

^31^P NMR OPLS-DA 1+1 68 16 0.405 0.271 0.018 8.84 × 10^−1^

LC-MS (+) OPLS-DA 1+2 70 66 0.513 0.527 0.346 1.16 × 10^−4^

LC-MS (-) OPLS-DA 1+1 70 54 0.302 0.430 0.131 5.44 × 10^−1^

**Comparison of ERG-positive PC samples with to ERG-negative PC samples.**

^1^H HR MAS NMR OPLS-DA 1+2 60 174 0.282 0.775 0.528 2.66 × 10^−7^

^1^H NMR OPLS-DA 1+2 70 103 0.401 0.598 0.142 1.28 × 10^−1^

^31^P NMR OPLS-DA 1+2 68 16 0.531 0.366 0.159 9.05 × 10^−1^

LC-MS (+) OPLS-DA 1+3 70 66 0.569 0.713 0.373 2.31 × 10^−4^

LC-MS (-) OPLS-DA 1+2 70 54 0.405 0.456 0.045 8.12 × 10^−1^

^a^All models are two-class OPLS-DA models.

^b^Comp: The number of predictive model components followed by the number of orthogonal model components.

^c^N samples: The number of samples the model is based on.

^d^N variables: The number of variables the model is based on.

^e^R^2^X: The amount of variation in X explained by the model, R^2^Y: The amount of variation in Y explained by the model, Q^2^: The amount of variation in Y predicted by the model.

^f^CV-ANOVA: p-value based on cross-validated scores showing the degree of significance for the separation.
