# Supplementary material for: Comprehensive metabolomics analysis of prostate cancer tissue in relation to tumor aggressiveness and TMPRSS2-ERG fusion status
Source: BMC Cancer. 2020 May 18;20:437. doi: 10.1186/s12885-020-06908-z (PMC7236196; doi:10.1186/s12885-020-06908-z)
Supplement: Supplementary file 8 — Additional file 8: Table S3. Detected proteins in β-oxidation and purine pathways. [file 12885_2020_6908_MOESM8_ESM.docx]

**Table S3.** Detected proteins in β-oxidation and purine pathways.

|  | Name | Pathway | ERG-positive PC vs ERG-negative PC | |
| --- | --- | --- | --- | --- |
|  |  |  | Direction of change | *p*-value* |
| 1 | ACSL1 | β-oxidation | ↓ | 0.022 |
| 2 | CPT1 | β-oxidation | ↑ | 0.021 |
| 3 | SLC25A20 | β-oxidation | ↑ | 0.493 |
| 4 | CPT2 | β-oxidation | ↓ | 0.018 |
| 5 | ACAD10 | β-oxidation | ↓ | 0.846 |
| 6 | ECHS1 | β-oxidation | ↓ | 0.171 |
| 7 | HADH | β-oxidation | ↓ | 0.050 |
| 8 | ACCA2 | β-oxidation | ↑ | 0.691 |
| 9 | HADHB | β-oxidation | ↓ | 0.789 |
| 10 | ACADM | β-oxidation | ↑ | 0.706 |
| 11 | ACCA1 | β-oxidation | ↓ | 0.220 |
| 12 | ACOX1 | β-oxidation | ↓ | 0.144 |
| 13 | ACOX3 | β-oxidation | ↓ | 0.087 |
| 14 | EHHADH | β-oxidation | ↓ | <0.0001 |
| 15 | ACADS | β-oxidation | ↓ | 0.661 |
| 16 | ACADL | β-oxidation | ↓ | 0.312 |
| 17 | ACADSB | β-oxidation | ↓ | 0.439 |
| 18 | HSD17B4 | β-oxidation | ↓ | 0.714 |
| 19 | ACAD9 | β-oxidation | ↓ | 0.617 |
| 20 | PNP | Purine | ↓ | 0.610 |
| 21 | HPRT1 | Purine | ↓ | 0.613 |
| 22 | ADA | Purine | ↑ | 0.152 |
| 23 | APRT | Purine | ↓ | 0.018 |
| 24 | NT5E | Purine | ↑ | 0.024 |
| 25 | ADSL | Purine | ↑ | 0.055 |
| 26 | AMPD3 | Purine | ↑ | 0.048 |
| 27 | AMPD2 | Purine | ↑ | 0.142 |
| 28 | ADSSL1 | Purine | ↓ | 0.772 |
| 29 | GDAP1 | Purine | ↓ | 0.462 |
| 30 | GDAP2 | Purine | ↑ | 0.396 |

* nonparametric t-test
